# Investigating the Impact of Amylopectin Chain-Length Distribution on the Structural and Functional Properties of Waxy Rice Starch

**DOI:** 10.3390/foods14234130

**Published:** 2025-12-02

**Authors:** Waqar ul Zaman, Zainab Ijaz, Muhammad Yousaf Nadeem, Shibbir Ahmed, Enpeng Li

**Affiliations:** 1 Jiangsu Key Laboratory of Crop Genomics and Molecular Breeding/Key Laboratory of Plant Functional Genomics of the Ministry of Education/Jiangsu Key Laboratory of Crop Genetics and Physiology, Agricultural College of Yangzhou University, Yangzhou 225009, China; dh18001@stu.yzu.edu.cn (W.u.Z.); dh18019@stu.yzu.edu.cn (Z.I.); 008625@yzu.edu.cn (M.Y.N.); 008627@yzu.edu.cn (S.A.); 2 Jiangsu Co-Innovation Center for Modern Production Technology of Grain Crops, Yangzhou University, Yangzhou 225009, China; 3 Jiangsu Key Laboratory of Crop Cultivation and Physiology, Agricultural College of Yangzhou University, Yangzhou 225009, China

**Keywords:** chain-length distribution, pasting properties, gelatinization, in vitro digestibility, structure–function relationship

## Abstract

This study investigated how amylopectin (AP) chain-length distribution (CLD) influences the structural and functional properties of ten waxy rice varieties through a multiscale analysis. The fitting CLD parameters revealed that varieties such as YN12 and GMN2 contained the highest proportions of intermediate chains (h_iii_~14.5~10.2) and long chains (h_v_~0.552~0.477), resulting in higher gelatinization temperatures, including onset temperatures (T_o_~60.2~66.4 °C), temperature peak (T_p_~72.0~72.2 °C), and the conclusion temperatures (T_c_~80.8~79.4 °C). Sample YN12 exhibited the lowest breakdown viscosity (BDV~748 cP), indicating higher thermal stability and stable pasting behavior. All of the samples showed relative crystallinity (Rc%) ranging from 19.3 to 22.8% with lamellar spacing of 8.8~9.4 nm. Sample YN12 exhibited the lowest enzymatic digestion rate (*k*~1.48 × 10^−2^ min^−1^) and the highest resistant starch (*C_res_*~9.81%), reflecting restricted enzyme accessibility. In contrast, sample SN9714 and ZN106, characterized by a higher proportion of shorter branches in the long-chain region of AP CLD (β_vi_~3.75~2.53), exhibited greater BDV (1407 cP) and faster digestion kinetics (*k*~1.90 × 10^−2^ min^−1^). The Rc% (22.83%) of NGXN was positively correlated with gelatinization enthalpy (ΔH~14.8 J/g). These findings highlight the pivotal role of AP CLDs in determining the structural, thermal, pasting, and digestive properties of waxy rice starch, offering molecular-level guidance for the development of rice-based ingredients with tailored functional characteristics.

## 1. Introduction

Rice is a major crop in China and an essential food product in Asian countries. The grain quality of rice is the primary focus for breeders worldwide aiming to improve living standards. Rice quality can be evaluated based on milling performance, granule morphology, cooking behavior, sensory attributes, nutritional value, and hygienic characteristics. Rice grain quality is primarily determined by its sensory and nutritional properties for consumers. Rice grain is composed of numerous components; among them, starch is the main and largest component, which occupies approximately 80% of the rice grain. Waxy rice (also known as glutinous rice) is considered a premium variety because when cooked it transforms into a sticky material. The dry weight of polished rice contains about 80% starch content. Waxy rice is mostly amylopectin (AP) and contains a very small amount (0–2%) of amylose (AM) [1]. Waxy rice starch has distinct properties, making it widely used in various industries, including pharmaceutical, food, industrial chemical, cosmetics, and biodegradable material sectors.

Starch primarily consists of AM and AP. AM forms a linear chain linked by α-(1–4) glycosidic bonds, whereas AP forms branched chains linked by α-(1–6) glycosidic bonds [2]. Starch is an important source of calories for humans and also serves as a key factor in evaluating the eating and cooking quality (ECQ) as well as the nutritional value of rice. AP has a highly branched network with shorter chains and a higher molecular weight, whereas AM contains fewer branches, longer chains, and a lower molecular weight compared to AP. The starch granules exhibit a semi-crystalline morphology primarily associated with a higher proportion of AP, which provides a firmer structure [3] and is reflected in molecular characteristics such as relative crystallinity and lamellar thickness. The molecular structure of AP is a key factor contributing to the diversity among waxy rice starches, influencing their thermal properties [4]. Research on starch digestibility and pasting properties has largely focused on whole starches containing both AM and AP. In such cases, structural variations in AM may mask the specific influence of AP. To better understand the role of AP, starches with minimal AM are more appropriate for evaluation. Waxy starch, which is composed of more than 98% AP, provides a suitable model for this purpose [5].

The structure–function relationship of AP has been extensively studied in various starch systems [6,7]. Previous research has established that a higher abundance of long AP chains is strongly correlated with enhanced paste stability and elevated gelatinization temperatures [8]. Conversely, a higher proportion of short chains is associated with increased swelling power and greater susceptibility to enzymatic hydrolysis [9]. However, a significant limitation of many existing studies lies in their reliance on normal starch varieties containing both AM and AP. In such systems, the pronounced influence of AM, particularly its tendency to form lipid complexes and undergo retrogradation, may obscure the specific contributions of AP chain-length distributions (CLDs) to critical functional properties, including pasting behavior and digestibility.

CLDs within AP add complexity to its role in gelatinization. Shorter chains promote faster swelling and disruption of the granule structure during heating, whereas longer chains help stabilize starch granules, leading to variations in gelatinization rates and the final viscosity of starch pastes during cooking [10]. This is especially important in food processing and meal preservation, where modifying starch properties can improve cooking performance and enhance sensory qualities, such as texture and viscosity. This intricate interaction impacts both the culinary and nutritional qualities of starchy foods, playing a significant role in consumer preference and overall health benefits [11]. Gelatinization enthalpy, a thermodynamic measure indicating the energy absorbed during the transformation from the crystalline to the amorphous phase, shows a strong association with the proportion of long AP chains [12].

The variations in gelatinization behavior, governed by AP CLDs, not only affect the functional properties of starch during cooking but also have significant implications for its digestibility and nutritional impact. The rapid digestion of starch-rich foods has been linked to several non-communicable diseases, including obesity and type 2 diabetes, whose prevalence has increased significantly over the past five decades in both developed and developing regions. In contrast, consumption of slowly digestible starch has been shown to attenuate postprandial glucose spikes, thereby contributing to reduced cardiovascular risk and the prevention of type 2 diabetes [5]. Digestibility and pasting properties are closely linked to certain characteristics of AP CLDs. Previous studies have reported that a lower proportion of short AP chains is linked to rapid digestible starch (RDS), while a higher proportion of short chains is related to slowly digestible starch (SDS) and resistant starch (RS). Additionally, medium and long AP chains are associated with RDS and SDS fractions [13]. Furthermore, Rapid Visco-Analyzer profiles have shown a negative correlation between the proportions of long AP chains and breakdown viscosity, whereas the short AP chains showed a positive correlation with breakdown viscosity [14]. The majority of studies on starch structure and digestibility have examined whole starches that contain both AM and AP, which may confound the distinct effects of AP due to variations in AM content. Thus, because waxy rice starch contains only a very small amount of AM, it is particularly useful for investigating the impact of AP-dominated starches on digestibility and pasting characteristics without interference from the higher AM content found in regular starch [15].

The aim of this research was to analyze the structural and functional properties of waxy rice starches, focusing on the connection between AP CLDs and their influence on starch digestibility, pasting, and other functional characteristics. Specifically, the study examined how the AP CLD affects starch digestibility in cooked rice and how this contributes to a slower digestion rate. Ultimately, the findings are expected to provide deeper insights into the role of AP structural features in determining starch functionality, contributing to the development of rice varieties with improved processing and quality attributes.

## 2. Materials and Methods

### 2.1. Materials

A total of ten different waxy rice varieties were obtained from the Zhenjiang Agricultural Science Research Institute. A milling machine (Scino, CT410, FOSS, Hagersten, Sweden) was employed to mill the rice grains, and milled grains were polished using a laboratory rice polisher (Pearlest, Kett, Tokyo, Japan) and then filtered through a 100-mesh screen. Protease (type XIV) from *Streptomyces griseus* was purchased from Sigma-Aldrich Chemical Co., (St. Louis, MO, USA). A total starch (AA/AMG) Assay kit and isoamylase from *Pseudomonas* sp. were purchased from Megazyme International, Ltd. (Bray, Co., Wicklow, Ireland). Amyloglucosidase (E-AMGDF) was obtained from Megazyme International, Ltd. (Bray, Co., Wicklow, Ireland), and 8-aminopyrene-1,3,6, trisulfonic acid (ATPS-M) was obtained from Beckman (Brea, CA, USA). Dimethyl sulfoxide (DMSO, GR grade) was obtained from Merck Co., Inc. (Darmstadt, Germany). Pullulan standards with peak molecular weights ranging from 342 to 2.35 × 10^6^ Da were purchased from Polymer Standards Service (PSS, Mainz, Germany). All other chemical reagents were used directly without additional purification.

### 2.2. Total Starch and Crude Protein Content

The total starch content was determined using the Total Starch Kit (K-TASA, Megazyme, Wicklow, Ireland) following a previously described method with minor modifications [16]. Waxy rice flours samples (20 mg) were mixed with 200 μL of absolute ethanol to remove the lipid residues. Thermostable α-amylase (in MOPS buffer, pH 6.5) and amyloglucosidase (in sodium acetate buffer, pH 4.5) were then used to hydrolyze starch into glucose. The obtained glucose solution was treated with glucose oxidase–peroxidase (GOPOD) reagent, which specifically reacts with glucose to produce a colored product, allowing for precise and selective quantification of glucose released during starch hydrolysis. The absorbance was measured using a spectrophotometer (Model F-4600, Hitachi, Tokyo, Japan). The starch content was calculated based on the glucose concentration using a conversion factor of 0.9.

A Kjeldahl apparatus (Omnilab, Bremen, Germany) was used to determine the nitrogen content of the samples. The crude protein content was calculated by multiplying the nitrogen content by a conversion factor of 5.95, corresponding to the nitrogen–protein conversion factor [17].

### 2.3. Scanning Electron Microscopy

Scanning electron microscopy (SEM) analysis was performed using an S4800-II instrument (Hitachi, Tokyo, Japan), as described previously [18]. Rice flour was mounted on an aluminum stub covered with double-sided carbon adhesive tape and then sputter-coated with a 10 nm layer of gold using an Eiko IB-3 ion sputter coater (Eiko Engineering Co., Ltd., Hitachinaka, Japan) under vacuum. All images were captured at an accelerating voltage of 10 kV at room temperature.

### 2.4. Size-Exclusion Chromatography

The starch was isolated from waxy rice flour using a previously described method [19]. A homogeneous mixture (2 mg/mL) was prepared by dissolving the extracted starch (4 mg) in dimethyl sulfoxide (DMSO) containing 0.5% (*w*/*w*) lithium bromide (LiBr). The size distributions of the fully branched starch were obtained using an LC-20 CE Shimadzu SEC system (Shimadzu Corporation, Kyoto, Japan) mounted with analytical columns GRAM precolumn, GRAM 30, and GRAM 3000 (Polymer Standard Services, Mannheim, Germany) in series and a Shimadzu RID-10A differential refractive index (DRI) detector, as described elsewhere [20]. The precolumns were 50 mm long, and the two analytical columns were 300 mm long. The particles of all of the columns had individual pore sizes (10 μm). The hydrodynamic radius (*R_h_*) was converted from elution time using Mark–Houwink parameters (including K = 0.0002427 dL g^−1^ and α = 0.6804) with pullulan standards (peak molecular weights varying from 342 to 2.35 × 106 Da, PSS) during calibration [21]. The SEC weight distribution *w*_br_(log*R_h_*) represents the weight fraction of branched starch molecules as a function of their *R_h_*, and size-exclusion chromatography (SEC) data are presented as the SEC weight-based distribution *w*_br_(log*R_h_*) [21].

### 2.5. Fluorophore-Assisted Carbohydrate Electrophoresis and Model Fitting

The isolated starches were debranched with isoamylase and labeled with APTS (8-aminopyrene-1,3,6-trisulfonic acid) according to the previously described method [22]. Labeled starch samples were then analyzed with an MDQ + FACE (fluorophore-assisted carbohydrate electrophoresis) system (Beckman, Coulter, Brea, CA, USA), fitted along with a solid-state laser-induced fluorescence (LIF) detector and an argon-ion laser as the excitation source. The degree of polymerization DP (*X*) was directly given by the FACE system, and the CLDs of starch branches *N_de_* (*X*) were plotted against DP (*X*).

AP CLDs were fitted using a model developed in a previous study [23]. The model assumes that AP CLDs are regulated by distinct enzyme sets, each comprising starch synthase (SS) and starch branching enzyme (SBE). Within each single set, a β value and an h value are defined: the β value represents the average chain length of each set, while the h value represents the number of chains in each set.

### 2.6. X-Ray Diffraction Analysis

A powder X-ray diffractometer (XRD) (D5005, Bruker AXS, Karlsruhe, Germany) was employed to analyze the crystallinity of starch granules. The XRD equipment was operated at 40 kV voltage and a 40 mA current. To maintain uniform moisture content, all of the samples were stored in a desiccator containing a saturated NaCl solution for 48 h prior to analysis. A glass sample holder was used to tightly pack the samples. Diffractograms were recorded over a diffraction angle at 2θ ranging from 4° to 40°, with a step interval of 0.013° and a scan rate of 3°/min. A baseline was constructed to represent the amorphous scattering background. For comparative purposes across the sample set, a consistent protocol was applied: a B-spline baseline was fitted between two fixed anchor points at approximately 5° and 40° 2θ for all samples. This baseline was then subtracted from the raw diffraction data. The total area under the resultant curve (At) from 5° to 40° 2θ was integrated, representing the total scattered intensity. The relative crystallinity (Rc%) was determined using Origin 2021 software according to the following equation:

(1) $$Rc\left(\%\right)=\sum_{i=1}^{n}\frac{AUCi}{AUCt}$$

where *AUCi* is the area under each crystalline peak with an index of *i* and *AUCt* is the total area of the diffraction pattern [24].

### 2.7. Small Angle X-Ray Scattering

Starch samples (100 mg) were mixed with 300 μL of distilled water in a 2 mL round-bottom centrifuge tube and allowed to equilibrate overnight at room temperature. The mixture was centrifuged at 4000× *g*, and the supernatant was discarded. The lamellar repeat distance (D) was determined using small-angle X-ray scattering (SAXS) with a Bruker Nanostar instrument (Bruker AXS Inc., Karlsruhe, Germany) equipped with a multilayer focused Cu Kɑ X-ray source. Adobe Photoshop (PS 2023) and Origin (Origin Pro 2021) were used to evaluate the raw data. The D, representing the average thickness of the semi-crystalline lamellae, was calculated using the Wolf–Bragg formula according to following equation:

(2) $$D=\frac{2\pi }{Smax}$$

where *D* represents the Lamellar repeat distance and *S_max_* is scattering vector at peak maximum.

### 2.8. Rapid Visco Analyzer

A Rapid Visco Analyzer (RVA-Tec Master, Perten, Stockholm, Sweden) was used to examine the pasting properties of waxy rice varieties according to previously published studies [25]. Briefly, 25 g of distilled water was added to an aluminum canister, followed by 3 g of accurately weighed waxy rice flour to ensure that the resulting slurry was completely dispersed. It was manually stirred with a plastic paddle before placing the canister in the instrument. After 1 min at 50 °C, the slurry was heated to 95 °C at a rate of 12 °C/min, kept for 2.5 min then cooled to 50 °C for 3.8 min, and then retained there for 3 min. During the first 10 s, the speed of rotation was adjusted to 960 rpm, and for the remaining test it was kept at 160 rpm.

### 2.9. Differential Scanning Calorimetry

The gelatinization and retrogradation properties were analyzed using differential scanning calorimetry (DSC) (Netzsch DSC 214 Polyma, Selb, Germany) following the method described [26]. Briefly, waxy rice flour (5 mg) was mixed with distilled water at a ratio of 1:3 (*w*/*v*), placed into a concave aluminum pan sealed hermetically, and allowed to equilibrate for 12 h. An empty aluminum pan served as the reference. The prepared samples were heated from 20 °C to 110 °C at a rate of 10 °C/min. Proteus analysis software (v 1.8.2) 2023 (Labcenter Electronics Ltd., Grassington, UK) was used to determine thermal parameters, including onset temperature (T_o_), peak temperature (T_p_), conclusion temperature (T_c_), and gelatinization enthalpy (ΔH).

For the retrogradation, the sample pans after gelatinization were stored at 4 °C for seven days and the stored samples were reheated from 20 °C to 110 °C at a rate of 5 °C/min, using the same empty aluminum pan as a reference [27].

### 2.10. In Vitro Starch Digestibility and Model Fitting

The in vitro starch digestion assay was performed using a previously reported method with minor modifications [28]. Waxy rice flour (100 mg) was suspended in 2 mL of distilled water and cooked in a boiling water bath for 30 min then cooled in a 37 °C water bath. Then, 8 mL of the digestion enzyme solution (containing 2.5 μL of amyloglucosidase and 0.05 mg of pancreatin in 0.2 M sodium acetate buffer pH 6.0) was added to the cooked rice samples. Aliquots (0.1 mL) were withdrawn at various time intervals (0, 5, 10, 15, 20, 30, 45, 60, 90, 120, and 180 min) and immediately mixed with 0.9 mL of absolute ethanol to terminate the enzyme reaction. After centrifugation at 3000 rpm for 10 min, the absorbance of the supernatant was measured using a spectrophotometer at 510 nm after reaction with GOPOD reagent. Glucose concentrations were determined from a standard calibration curve prepared with known glucose solutions.

The digestion kinetics were analyzed by fitting the data to a first-order kinetics model [29]. For regions following first-order kinetics, the digestion rate constant (*k*) and the undigested starch (*C*_res_) were calculated using non-linear least-squares fitting (NLLS) of the following equation:

(3) $$Ct=\left(Co-Cres\right)e^{-kt}+Cres$$

where *C_t_* represents the starch proportion that remains undigested at time *t*, *Co* denotes the initial quantity of digestible starch at t = 0, *C_res_* indicates the remaining undigested starch at an infinite time, and *k* is the rate constant for starch digestion.

### 2.11. Statistical Analysis

All data are presented as means ± standard deviation (SD). Statistical analyses were performed using SPSS software (version 22.0; IBM Corp., Armonk, NY, USA). A one-way analysis of variance (ANOVA) was applied to assess differences among groups, followed by post hoc comparisons using the Least Significant Difference (LSD) and Duncan’s multiple range tests. Differences were considered statistically significant at *p* ≤ 0.05.

## 3. Results and Discussion

### 3.1. Chemical Composition

The starch content of the ten waxy rice samples varied from 81.93% to 88.01%, with WGN1 showing the highest starch content and GMN2 the lowest (Table 1). This result is consistent with previous studies on waxy rice starch [30]. The protein content of the ten waxy rice samples ranged between 5.97% and 7.21%, which is lower than those reported for common rice varieties [31]. Low protein content in rice is generally associated with better eating and cooking properties [31]. Proteins can affect starch functionality by partially restricting granule swelling during heating or by forming complexes with AP chains, resulting in a firmer texture [32]. However, because all samples exhibited similar protein content, the effect on functional properties is expected to be minimal, and fluctuations are primarily controlled by intrinsic differences in AP CLDs.

### 3.2. Starch Granule Morphology

**Figure 1:**
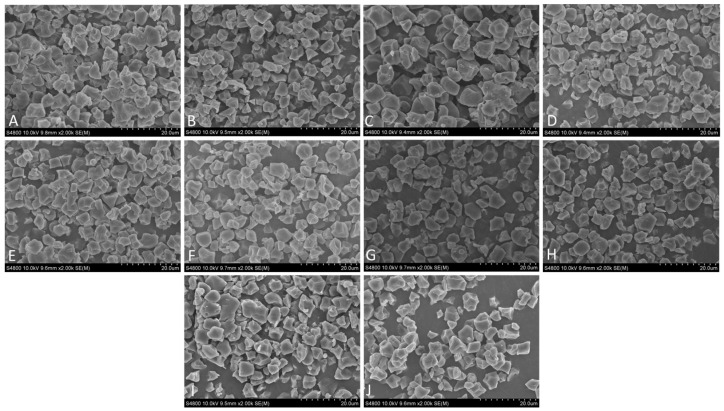
Scanning electron micrographs of rice flours from ten waxy rice varieties, (**A**) NGXN, (**B**) ZN19, (**C**) GMN2, (**D**) SN9714, (**E**) CJN6, (**F**) ZN106, (**G**) ZN65, (**H**) THN, (**I**) WGN1, (**J**) YN12. The scale bar is labeled as ‘um’ in the image as generated by the instrument and corresponds to micrometers (µm).

Scanning electron micrographs of the isolated starch granules from the 10 waxy rice varieties are presented in Figure 1. SEM analysis revealed that all isolated starch granules exhibited typical polyhedral shapes with sharp edges and smooth surfaces, consistent with the reported morphology of waxy rice starch [33]. The granule size varied among varieties, ranging from approximately 3 to 10 μm [34]. Notable differences in granule size were observed among the varieties. The smooth surfaces and lack of visible pores indicate well-preserved structural integrity in their native state. The size distribution varied, with some varieties like NGXN and GMN2 consisting predominantly of larger, uniform polygonal granules with sharp edges and smooth surfaces [33]. In contrast, varieties like SN9714 and ZN19 exhibited smaller granule sizes with slightly rough surfaces. Varieties like CJN6, ZN106, ZN65, and THN contained both large and small granules with irregular sizes and more compact structures [35]. The granule size distribution directly influences the surface area to volume ratio, which governs water absorption capacity and swelling behavior during heating. The overall structural integrity observed across all samples provides a morphological explanation for the differences in pasting properties, higher thermal stability, and stable pasting behavior.

### 3.3. Molecular Size Distribution of Fully Branched Waxy Rice Starches

**Figure 2:**
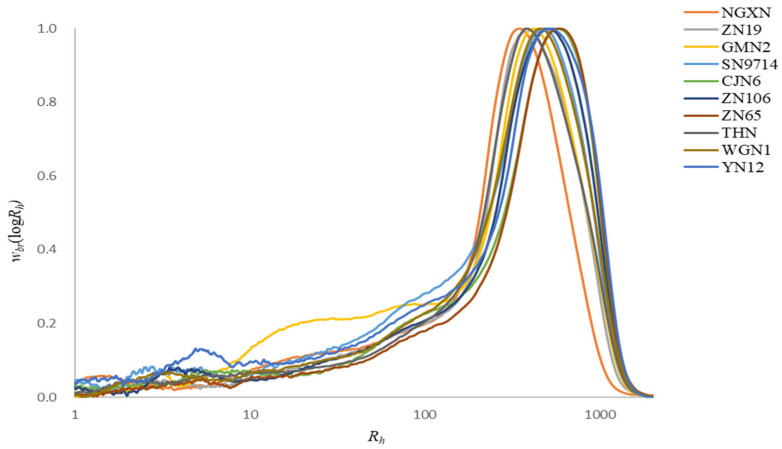
SEC weight distributions, *w*_br_(log*R_h_*), of fully branched starches from ten waxy rice varieties; all distributions were normalized to the peak maximum.

The molecular weight size distribution, *w*_br_(log*R_h_*), of whole (branched) starch molecules from ten different waxy rice varieties is presented in Figure 2. The chromatograms revealed a single major peak, with a distinct maximum at an *R_h_* of approximately 1000 nm, corresponding to AP. The highest average *R_h_* values were observed in samples ZN65 and CJN6 in Table 2, indicating that these waxy rice samples have larger AP molecular sizes compared to the others. Previous studies have reported that AP, which is predominant in waxy rice starch, generally possesses larger molecular sizes and contributes to functional properties, such as viscosity and gelatinization [36]. The larger molecular size of AP in ZN65 and CJN6 is likely a key factor influencing their functional performance during food processing, as larger molecules can entangle more easily and form stronger networks [37].

### 3.4. Structural Analysis of Amylopectin Chain-Length Distribution

**Figure 3:**
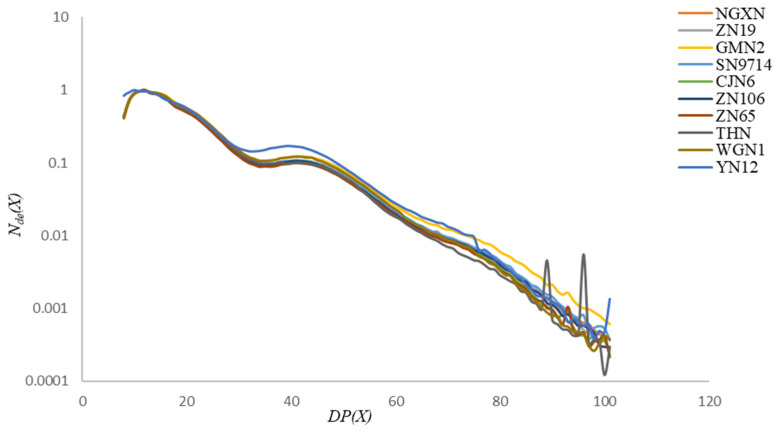
The CLDs of ten debranched starch samples measured through FACE, a high-resolution technique that accurately quantifies AP CLDs. The *Y*-axis shows the debranched number distribution *N*_de_(*X*), and the *X*-axis represents the degree of polymerization DP(*X*).

AP CLDs of ten different waxy rice starches are shown in Figure 3. All distributions were normalized to their maximum values. The CLDs exhibited several distinct features: a first shoulder at approximately DP~12~14, a second broad shoulder spanning DP~33~60, and a third shoulder in the DP~63~96 range. The first shoulder at DP~12~14 corresponds to shorter chains that form the single crystalline lamella. The second and third shoulders represent longer chains spanning multiple crystalline lamellae, which are critical for stabilizing the overall crystalline architecture of the starch granule [37]. This finding is supported by previous studies analyzing the effects of environmental warming on rice flour, which reported significant alterations in the AP CLDs [38]. Research indicates that the relative abundance of B1 chains is crucial for determining the gelatinization properties of starch, with important implications for cooking and processing behaviors. The outermost chains, recognized as A and B1 chains, are primarily located in the crystalline regions and play a key role in the formation of double helices, which confer the distinctive lamellar structure of starch granules [39]. Furthermore, the interior chains, such as B2 and B3 chains, are located within the amorphous domains and are essential for granule swelling and solubility [40].

### 3.5. Biosynthesis Model for Amylopectin CLD Fitting

The model fitting of AP CLDs was performed using a biosynthesis-based model derived from enzymatically debranched starches via FACE, as described elsewhere [41]. The biosynthesis model posits that each enzyme set contributes primarily, though not exclusively, to specific regions of AP biosynthesis, consisting of one or two isoforms of starch synthase (SS), starch branching enzyme (SBE), and debranching enzyme (DBE). This model suggests that distinct enzyme sets, each primarily comprising specific isoforms of SS, SBE, and DBE, are responsible for synthesizing different regions of the AP molecule, with their interaction governing the overall CLDs [42]. Two core parameters, h_i_ and β_i_, are involved in this biosynthesis model, where h_i_ defines the relative number of chains in AP enzyme set i and β_i_ represents the activity ratio of SBE to SS within that specific enzyme set. The values of eight parameters (β_i_, β_ii_, β_iii_, β_iv_, β_v_, β_vi_, h_iii_, and h_v_) describing AP biosynthesis were obtained by fitting the CLDs to the AP biosynthesis model [41]. The data regarding the AP fitting parameters are presented in Table 2.

The h_iii_ and h_v_ values for sample YN12 and GMN2 were the highest, indicating that they possess the highest number of medium and long AP chains [42]. The β_i_ values for CJN6 and ZN65 were the highest (10.3~9.88), demonstrating a greater density of shorter chains in the AP short-chain region. The β_iii_ values were highest for samples YN12 (8.44) and WGN1 (6.96), indicating a greater number of short branch chains in the AP intermediate-chain region. Samples THN and WGN1 showed the highest β_v_ values (10.5~10.0), suggesting that they contain more short branches in the AP long-chain region. These variations in fitted parameter values across the different waxy rice varieties highlight the genetic diversity in starch biosynthetic enzyme activity, which ultimately dictates the CLD of AP and, consequently, the functional properties of the starch.

### 3.6. Relative Crystallinity

**Figure 4:**
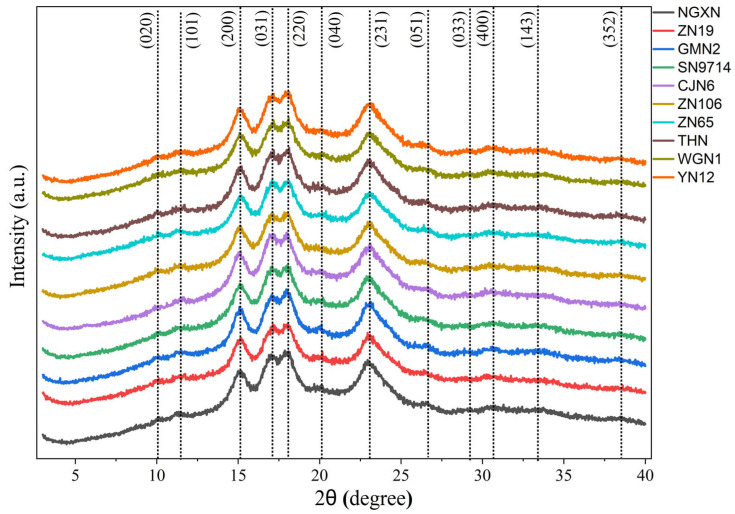
X-ray diffraction patterns of ten different waxy rice flours.

The X-ray diffraction patterns of ten waxy rice varieties are illustrated in Figure 4. The diffraction peaks observed at 2θ of 15°, 17°, 18°, and 23° exhibited relatively higher intensity compared with the other peaks. Other additional peaks were observed at 2θ of 10°, 11.4°, 20.1°, 26.6°, 29.1°, 30.6°, 33.4°, and 38.4°. The diffraction peaks of waxy rice flour were indexed according to a previous study by Martinez-Munoz et al. [43] for A-type cereal starches. The Rc% of the ten waxy rice varieties ranged from 19.29% to 22.83% (Table 3). All waxy rice samples exhibited diffraction patterns corresponding to starch nanocrystals with an orthorhombic crystal structure typically classified as A-type starch consistent with the findings of Rodriguez-Garcia et al. [44]. Sample NGXN showed the highest Rc% (22.83%), whereas the lowest was observed in sample CJN6 (19.29%). The NGXN exhibited a balanced amylopectin chain-length distribution, with a relatively higher proportion of short chains (β_i_~9.22, β_ii_~1.38) that effectively formed double helices within the crystalline lamellae. Meanwhile, the long chains of NGXN (β_v_~7.39, β_vi_~1.58) in AP CLD are longer, causing less disruption and allowing its abundant short chains to form a more perfect crystalline structure [10]. The high degree of visual similarity between the diffractograms is attributed to their fundamental biological similarity, including very low AM content and high AP content. NGXN showed the highest *R_c_*% (22.83%) and ΔH (14.8 J·g^−1^), confirmed by correlation results [45], indicating a more ordered and thermally stable crystalline structure among all of the tested varieties. The detailed XRD data is provided in the Supplementary Materials.

### 3.7. Lamellar Structure

SAXS was used to investigate the lamellar structure of ten waxy rice starches (Table 3). A clear primary scattering peak was detected at a scattering vector (qo) of around 0.06 Å^−1^, which is attributed to the periodic alternation between crystalline and amorphous lamellae within the AP structure. The position of this peak (q) determines the lamellar repeat distance and varies among different starch sources. The peak intensity depends on the electron density contrast between the crystalline and amorphous layers, which is related to the degree of structural order in the semi-crystalline region [2]. The I_max_ values representing the maximum scattering intensity varied across samples (244.44~558.58), indicating differences in electron density contrast or structural density. The S_max_ values (68~71) were comparatively consistent, suggesting similar periodic distances in the nanostructures, consistent with typical lamellar spacings reported for native starch granules.

The ΔS values, reflecting peak width, show variability (180.72~235.1), with broader peaks indicating less ordered or more heterogeneous structures. The D values (Bragg spacing) ranged from 8.80 to 9.38 nm. Among all waxy rice samples, ZN19 and NGXN showed the highest D values at 9.38 nm and 9.24 nm, respectively. The combined observations of variable peak intensities and consistent repeat distances suggest subtle differences in the degree of crystalline perfection rather than major changes in the lamellar architecture. This variation in organization within the semi-crystalline zones of the granules [46] is consistent with previous studies, which report that this characteristic scattering peak reflects an organized arrangement with repeat distances typically ranging from 9 to 10 nm in native starches [47].

### 3.8. Structural Correlation Analysis

**Figure 5:**
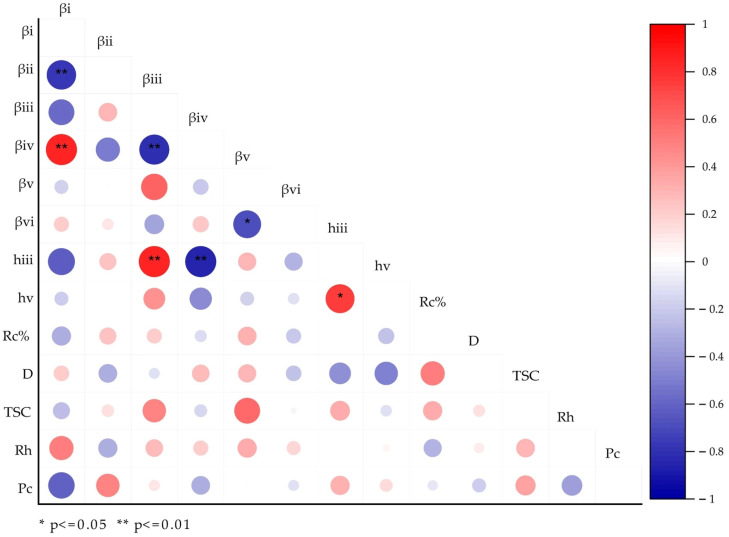
Pearson correlation between structural parameters. The stars (*) indicate statistically significant correlation. Red color indicates positive correlation; blue color indicates negative correlation.

The structural correlation analysis is presented in Figure 5. The β_i_ value showed a strong negative correlation with β_ii_, and β_iii_ exhibited a negative correlation with β_iv_. These results indicate that synthesis of short chains (mainly in the amorphous region) and long chains (mainly in the crystalline region) within the same region is competitive, consistent with the findings of Han et al. [48]. Furthermore, β_v_ revealed a negative correlation with β_vi_, which differs from the observation of Han et al., where no correlation was observed between β_v_ and β_vi_. This variation can be linked to the type of starch analyzed. In Han’s study, normal starch was investigated, in which granule-bound starch synthase (GBSSI) was not only involved in AM biosynthesis but also affected the biosynthesis of long AP chains (DP > 70). Conversely, the samples used in the current study were all waxy rice, which lacks GBSSI. Without the influence of GBSSI, the biosynthesis of long AP chains was carried out in a similar way to short and medium AP chains.

The value of h_iii_ showed a positive correlation with the h_v_ value, which reflects that the synthesis of medium and long AP chains is probably controlled by the same enzyme sets in waxy rice. Moreover, the value of h_iii_ also exhibited a positive correlation with β_ii_ and a negative correlation with β_vi_, which was not reported in former studies [48]. This result suggests that the amount of medium AP chains in waxy rice starch may be affected by its chain length (samples with longer chains tend to have a higher number of chains).

### 3.9. Pasting Properties

**Figure 6:**
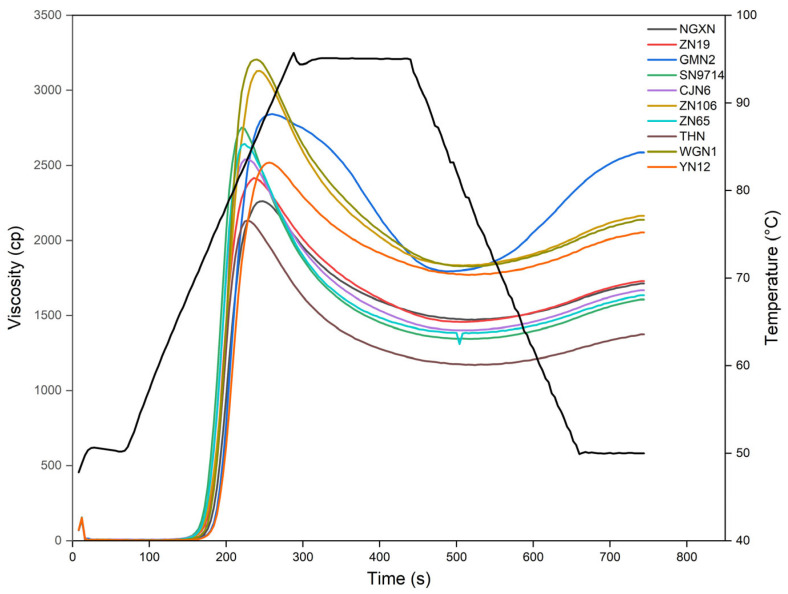
Rapid Visco Analyzer (RVA) curves of rice flours from ten waxy varieties. Lines in the figure correspond to the respective rice samples as indicated in the legend. The black line represents the temperature profile during RVA analysis.

The pasting characteristics of ten waxy rice flours were examined using a Rapid Visco Analyzer (RVA) and are presented in Table 4, with the corresponding pasting curves displayed in Figure 6. The pasting parameters were directly obtained from the RVA viscosity profiles using the instrument’s software during controlled heating, holding, and cooling cycles. Peak viscosity (PV), which reflects the maximum swelling of starch granules prior to rupture, ranged from 2134 to 3205 cP, with WGNI (3205 cP) and ZN106 (3130 cP) showing the highest PV and THN (2134 cP) and NGXN (2261 cP) the lowest. Trough viscosity (TV), representing the viscosity of the starch paste after granule rupture, ranged from 1171 to 1833 cP, with ZN106 (1833 cP) and WGN1 (1826 cP) showing the highest values and THN (1171 cP) the lowest. Breakdown viscosity (BDV), which indicates the degree of disintegration of swollen starch granules during heating [49], ranged from 748 to 1407 cP. SN9714 (1407 cP) showed the highest BDV, whereas YN12 (748 cP) exhibited the lowest. Final viscosity (FV), indicative of the viscosity of the cooled paste, varied from 1375 to 2587 cP, with NGXN (2587 cP) being the highest and THN (1375 cP) the lowest. Setback viscosity (SBV), which reflects the reassociation and retrogradation tendency of starch molecules during cooling, ranged from 204 to 792 cP, with NGXN showing the highest value (792 cP) and THN (204 cP) the lowest. Pasting time and pasting temperature represent the time and temperature required to reach peak viscosity. Pasting time (PT min) ranged from 3.54 to 4.27 min, while the pasting temperature (PT °C) varied from 70.95 to 74.90 °C.

WGN1 and ZN106, with higher proportions of short chains in both short- and long-chain regions (β_v_~10.0, β_vi_~2.53) in AP CLDs, formed a less stable crystalline structure, which increases water uptake and promotes extensive granule swelling, elevating the PV. Among the ten samples, YN12 showed the lowest BDV and the highest pasting time (PT min), revealing greater stability. This stability is attributed to the abundance of longer AP chains (h_v_), which form a stronger granular structure that is more resistant to swelling and rupture. In contrast, sample SN9714 showed the highest BDV and the lowest pasting temperature (PT °C) values, likely due to its shorter branches in the AP long-chain region (β_vi_~3.75).

Two important parameters, SBV and FV, reflect the recrystallization of AM and longer AP chains during starch paste cooling [49]. Among all of the waxy rice samples, GMN2 displayed the highest FV and SBV viscosity values (2587 cP, 792 cP) (Table 4), as it possesses a relatively high proportion of long chains. YN12, despite having higher proportions of intermediate (h_iii_) and long chains (h_v_), did not show the highest FV and SBV, because YN12 has a higher proportion of shorter branches in the long-chain region of AP CLDs. These RVA results align with previous studies revealing that waxy rice starches with a higher proportion of short branches generally exhibit lower PT °C and higher BDV [50]. Longer AP chains can form inter-chain associations during heating and cooling, reinforcing granule structure and contributing to higher paste stability, as reflected in lower BDV and longer PT min. In waxy rice starch, where amylose content is minimal, the reassociation of long AP chains becomes the dominant factor controlling retrogradation and paste viscosity. In previous research, starch with an abundance of longer AP chains showed higher retrogradation [14].

### 3.10. Thermal Properties

The thermal characteristics of waxy rice varieties were analyzed using DSC. The thermal parameters, including T_o_, T_p_, T_c_, and ΔH, are illustrated in Table 5. T_o_ ranges from 60.2 °C to 66.4 °C, and the highest T_o_ was observed in sample YN12 (66.4 °C) and the lowest in sample CJN6 (60.2 °C). T_p_ ranged between 68.2 °C and 72.2 °C, and the highest was observed in sample YN12 (72.2 °C) and the lowest in sample CJN6 (68.2 °C). T_c_ varied from 77.4 °C to 80.8 °C; sample YN12 (80.8 °C) represents the highest value, and minimum values were exhibited by samples CJN6 and ZN106 (77.4 °C for both samples). The ΔH ranged between 12.3 and 14.8 (J/g). The ΔH of sample NGXN was the highest at 14.8 (J/g) and the lowest in sample SN9714, which was 12.3 (J/g).

Sample YN12 showed the highest values (T_o,_ T_p_, T_c_) among all of the tested waxy rice starches. Sample YN12 had the highest medium (h_iii_) and long chains (h_v_) in AP CLD (Table 2), which provides more ordered structure in starch granules, thereby hindering the gelatinization process and leading to higher transition temperatures, as reported in previous studies [51]. The gelatinization temperature results in this study highlighted the distinctive thermal behavior of AP-rich starches and are consistent with previous findings, revealing that gelatinization temperatures of waxy rice starches fall within comparable ranges [52]. ΔH represents the energy required to melt the crystalline regions within the starch granule, which corresponds to nanocrystals with an orthorhombic structure. The description of gelatinization has been updated to indicate that enthalpy reflects the energy required for solvation and disruption of these orthorhombic nanocrystals during gelatinization. Among the ten samples, NGXN, with the highest Rc%, also exhibited the highest ΔH, 14.8 (J/g), indicating that the highest ΔH observed for sample NGXN is a direct consequence of its high crystallinity [53].

The T_o_, T_p_, and T_c_ for retrogradation among waxy rice starches ranged from 46.9 °C to 65.1 °C, 49.8 °C to 67.3 °C, and 57 °C to 69.2 °C, respectively. The ΔH varied from 0.1 to 0.3 J/g). AP facilitates the lower ΔH values observed during retrogradation due to its highly branched structure, which limits the efficient reassociation of glucose chains into ordered crystalline regions. The recrystallization of AP chains after gelatinization is primarily responsible for the retrogradation of starch, and the chain-length distributions have a strong influence on the physical changes that occur during this process. These observations were consistent with the findings of Velazquez et al. [54], who reported that the degree of crystallinity and rheological recovery during long-term retrogradation are determined by the molecular architecture of amylopectin and the packing of double helices formed by glucose chains into ordered structures. It should be noted that waxy rice starch, which contains very low amylose content, exhibits limited retrogradation; therefore, the ΔH values observed in DSC reflect this reduced recrystallization, which is consistent with previous studies on waxy starch [51].

### 3.11. In Vitro Starch Digestibility

**Figure 7:**
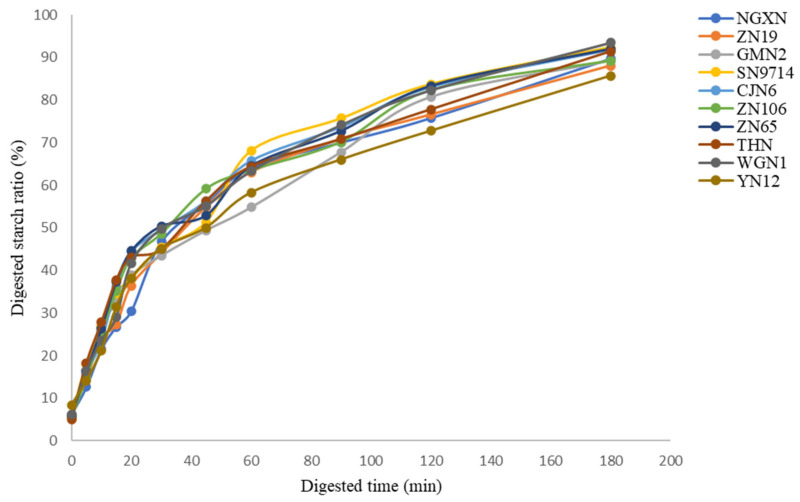
Digested starch ratio (%) curves of ten different waxy rice starches.

Starch digestion via an enzymatic process is an essential metabolic pathway that regulates postprandial blood glucose levels in humans. The rate and extent of starch digestibility in the small intestine have significant consequences for human health and nutrition [55]. The experimental digestograms of ten waxy rice varieties were obtained at different digestion time points, as demonstrated in Figure 7. The percentage of undigested starch (*C*_res_) and the digestion rate constant (*k*) indicate the extent and speed at which waxy rice starch is hydrolyzed into glucose during digestion. Among the waxy varieties, sample ZN106 revealed the highest digestion rate (*k*~1.90), suggesting highly digestible starch, while GMN2 (*k*~1.47) and YN12 (*k*~1.48) showed the lowest rates (Table 6). Previous research indicates that low AM starches, such as waxy rice starch, tend to exhibit higher digestibility compared to high AM starches, which may resist digestion because amylose can reassociate into tightly packed, enzyme-resistant structures during and after heating, thereby limiting enzymatic accessibility [56]. In this study, samples YN12 and ZN19 contained the highest *C*_res_ content, implying that these waxy starches may offer health benefits, such as lower glycemic impact and potential prebiotic effects. The distinctive CLDs and branching patterns of AP in these varieties appear to limit enzymatic accessibility, thereby modulating starch digestibility and the rate of glucose release [57].

### 3.12. Correlation Analysis Between Structural Parameters and Functional Characteristics

**Figure 8:**
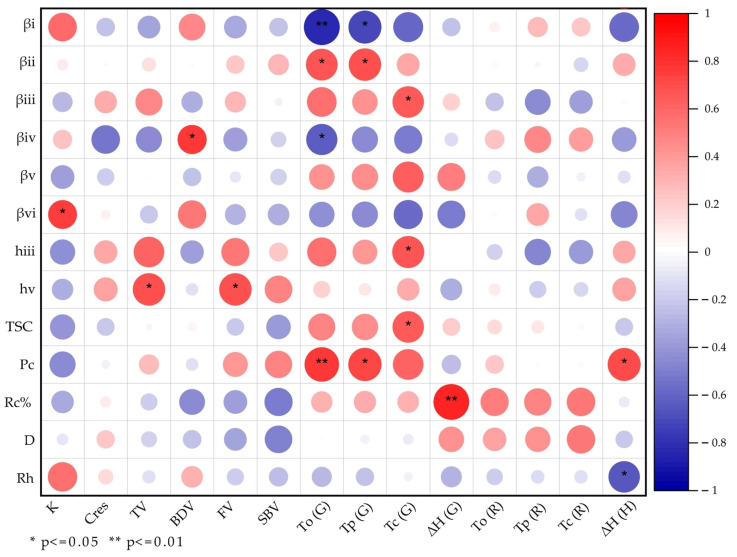
Pearson correlation analysis between structural attributes and functional properties of waxy rice varieties. The stars (*) indicate statistically significant correlations. Red color: positive correlation; blue color: negative correlation.

Pearson’s correlation analysis was conducted to evaluate the relationship between structural parameters and the functional characteristics of the waxy rice varieties (Figure 8). A positive correlation was observed between FV and SBV with the h_v_ parameter. FV and SBV were believed to be mainly affected by the amylose content (AC) in normal starch [58]. While in waxy starches, these two pasting parameters were controlled by the amount of AP long chains. This result indicated that FV and SBV were mainly controlled by the longest starch branches. The value of BDV showed a positive correlation, as BDV value calculation is based on PV and TV. Nevertheless, no correlations were observed between PV/TV and β_iv_, indicating that the BDV linkage may be a statistical artifact rather than a functional correlation.

The digestion rate (*k*) showed a positive correlation with the β_vi_ value, and because a larger β value represents a shorter chain length, this correlation indicates that shorter AP long chains could increase the digestion rate of waxy rice starch, which is consistent with various earlier findings [59,60]. Residual proteins may also affect starch digestibility by acting as physical barriers that limit enzyme access to AP chains. Nevertheless, the relatively small variation in protein content among the tested samples suggests that its impact on digestion kinetics is likely secondary compared to the influence of AP CLDs. This interpretation aligns with our correlation analysis, where protein content showed only a weak association with gelatinization temperatures and no significant correlation with *k*. Therefore, the observed functional differences can be primarily attributed to variations in AP structure rather than protein–starch interactions.

ΔH exhibited a positive correlation with Rc%. As waxy rice is mostly AP, it has a more uniform and ordered structure in comparison with non-waxy rice. As a result, an increase in the proportion of these ordered structures requires a higher energy barrier to melt, which leads to a higher observed gelatinization enthalpy [61]. Gelatinization temperatures, including T_o_, T_p_, and T_c_ reveal the extent of molecular order within the starch granules [62]. Previous studies have demonstrated that a higher relative content of shorter chains is typically linked to lower gelatinization temperatures because these chains are prone to forming less stable and double helices [63]. In our study, T_o_ and T_c_ showed a negative correlation with β_i_ and a positive correlation with β_ii_. β_i_ mainly refers to the length of A and B1 chains; the correlation results indicated that long A and B1 chains could lead to high onset and peak temperatures, which is consistent with former studies. Protein content was also found to have a positive effect on T_o_ and T_c_, suggesting that protein in waxy rice could suspend the gelatinization of AP. Additionally, T_c_ exhibited a positive correlation with β_iii_, h_iii_, and total starch content; this result indicated that the completion of gelatinization mainly depended on the total number of branches, especially the number of medium AP chains.

The molecular size of whole starch exhibited a negative correlation with the enthalpy changes of retrogradation, revealing that the larger AP molecules minimize the chances of recrystallization after gelatinization. Other retrogradation parameters showed no significant correlations with starch’s structural characteristics because the recrystallization of starch chains occurs randomly and the native starch structural parameters do not directly affect the retrogradation parameters. The correlation results suggest that waxy rice starch with low protein content and large molecular size may exhibit a stronger tendency to reassociate and recrystallize during storage, indicating enhanced retrogradation behavior.

## 4. Conclusions

This study investigated the influence of AP CLDs on the functional properties of ten waxy rice varieties. SEC and FACE analyses demonstrated distinct molecular size distributions and branching structures. XRD analysis verified that all waxy rice starches are A-type cereal starches. SEM analysis revealed that waxy starch granules had irregular polyhedral shapes with sharp edges and smooth surfaces. DSC measurements supported these structural findings. Varieties like YN12 and GMN2 with a higher proportion of long chains exhibited elevated gelatinization temperatures and lower breakdown viscosity, making them suitable for foods requiring thermal stability and stable pastes, such as canned, ready-to-eat, or frozen rice products. In contrast, varieties like SN9714 and ZN106, enriched in short AP chains, showed higher breakdown viscosities and faster in vitro digestion, indicating more readily disrupted granules and increased enzyme accessibility, which is advantageous for rapidly digestible foods and instant foods. Overall, these multiscale analyses demonstrate that AP CLDs are the central factor controlling the structural and functional properties of waxy rice starch, providing molecular-level criteria breeders can use to select or develop waxy rice varieties with targeted functional properties. As this work focused mainly on starch molecular structures, the absence of mineral and lipid profiling, components known to influence gelatinization, pasting, and amylose–lipid interactions, may limit complete interpretation. Incorporating these factors in future studies will enable a more comprehensive understanding of waxy rice’s starch behavior.

## Figures and Tables

**Table 1:** Basic information and chemical composition of ten waxy rice varieties.

Names	Sample Code	Total Starch Content (%)	Crude Protein Content (%)
Nangengxiangnuo	NGXN	83.35 ± 1.23 ^cde^	6.20 ± 0.08 ^de^
Zhennuo19	ZN19	85.13 ± 1.38 ^bcd^	7.18 ± 0.16 ^a^
Guangmingnuo2	GMN2	81.93 ± 0.50 ^e^	7.21 ± 0.09 ^a^
Shaonuo9714	SN9714	85.13 ± 1.53 ^bcd^	6.81 ± 0.15 ^b^
Chunjiangnuo6	CJN6	83.03 ± 0.58 ^de^	5.97 ± 0.28 ^e^
Zhenuo106	ZN106	82.39 ± 2.87 ^e^	6.18 ± 0.01 ^de^
Zhenuo65	ZN65	85.65 ± 0.43 ^abc^	6.46 ± 0.04 ^cd^
Taihunuo	THN	85.72 ± 0.81 ^abc^	6.69 ± 0.02 ^bc^
Wangengnuo1	WGN1	88.01 ± 1.16 ^a^	6.95 ± 0.06 ^ab^
Yannuo12	YN12	86.99 ± 1.12 ^ab^	6.84 ± 0.04 ^b^

Data are represented as means ± standard deviation. The same column values with different letters represent significant differences at *p* ≤ 0.05.

**Table 2:** Amylopectin structural fitting parameters of ten different waxy rice varieties.

Varieties	ꞵ_i_/10^−2^	ꞵ_ii_/10^−2^	ꞵ_iii_/10^−2^	β_iv_/10^−2^	ꞵ_v_/10^−2^	β_vi_/10^−2^	h_iii_/10^−2^	h_v_/10^−2^	*R_h_*
NGXN	9.22 ± 0.11 ^a^	1.38 ± 0.47 ^a^	6.01 ± 0.22 ^b^	2.48 ± 0.14 ^ab^	7.39 ± 0.35 ^a^	1.58 ± 0.45 ^ab^	7.92 ± 1.24 ^b^	0.32 ± 0.06 ^b^	317.84 ± 14.71 ^d^
ZN19	9.25 ± 0.71 ^a^	1.25 ± 0.12 ^ab^	5.94 ± 1.48 ^b^	2.46 ± 0.18 ^ab^	6.83 ± 0.38 ^a^	2.17 ± 0.39 ^ab^	7.87 ± 0.21 ^b^	0.31 ± 0.00 ^b^	363.75 ± 15.71 ^bc^
GMN2	8.68 ± 0.83 ^a^	2.15 ± 0.42 ^a^	6.26 ± 1.27 ^b^	1.80 ± 0.01 ^ab^	7.09 ± 1.12 ^a^	1.02 ± 0.64 ^b^	10.15 ± 0.07 ^b^	0.48 ± 0.09 ^ab^	323.70 ± 2.54 ^cd^
SN9714	9.13 ± 2.23 ^a^	2.11 ± 0.19 ^a^	5.95 ± 1.13 ^b^	2.39 ± 0.33 ^ab^	5.16 ± 0.28 ^a^	3.75 ± 0.81 ^a^	8.72 ± 1.39 ^b^	0.34 ± 0.12 ^b^	364.21 ± 16.91 ^bc^
CJN6	10.3 ± 0.01 ^a^	0.00 ± 0.00 ^b^	6.12 ± 0.00 ^b^	2.89 ± 0.00 ^a^	8.15 ± 0.00 ^a^	1.72 ± 0.00 ^ab^	8.19 ± 0.00 ^b^	0.35 ± 0.00 ^b^	406.31 ± 29.96 ^ab^
ZN106	8.47 ± 0.18 ^a^	1.12 ± 1.00 ^ab^	7.49 ± 1.57 ^ab^	1.27 ± 1.21 ^bc^	7.74 ± 1.22 ^a^	1.35 ± 1.33 ^b^	8.94 ± 0.47 ^b^	0.39 ± 0.02 ^ab^	396.55 ± 0.22 ^ab^
ZN65	9.88 ± 0.60 ^a^	1.57 ± 1.05 ^a^	6.46 ± 0.60 ^b^	3.08 ± 0.49 ^a^	9.20 ± 1.12 ^a^	1.81 ± 0.83 ^ab^	8.05 ± 0.76 ^b^	0.38 ± 0.02 ^ab^	427.61 ± 11.82 ^a^
THN	8.64 ± 1.27 ^a^	2.31 ± 0.31 ^a^	6.86 ± 1.12 ^ab^	1.81 ± 0.04 ^ab^	10.51 ± 7.21 ^a^	1.44 ± 2.04 ^b^	8.02 ± 1.53 ^b^	0.13 ± 0.17 ^b^	366.83 ± 28.93 ^b^
WGN1	9.09 ± 0.27 ^a^	1.53 ± 0.55 ^a^	6.96 ± 0.57 ^ab^	2.53 ± 0.93 ^ab^	10.04 ± 0.65 ^a^	1.47 ± 0.37 ^b^	9.72 ± 1.67 ^b^	0.33 ± 0.02 ^b^	379.59 ± 17.85 ^b^
YN12	8.29 ± 0.00 ^a^	1.85 ± 0.00 ^a^	8.44 ± 0.01 ^a^	0.37 ± 0.00 ^c^	9.77 ± 0.01 ^a^	1.38 ± 0.03 ^b^	14.50 ± 0.03 ^a^	0.55 ± 0.00 ^a^	382.84 ± 10.13 ^b^

β and h are fitting parameters; a higher β value represents short chains in each dominated set, and a larger h value means higher amounts of chains in each dominated set. *R_h_*: hydrodynamic radius. Data are used with replications represented as means ± standard deviation. Different superscript letters within the same column indicate significant differences at *p* ≤ 0.05.

**Table 3:** Relative crystalline and lamellar repeat distance of ten different waxy rice varieties.

Varieties	XRD	SAXS			
	Rc (%)	I_max_	S_max_ × 10^−3^	∆S × 10^−4^	D (nm)
NGXN	22.83 ± 1.19 ^a^	558.58	68.00	195.10	9.24
ZN19	20.83 ± 1.38 ^ab^	363.30	66.91	235.10	9.38
GMN2	19.76 ±1.06 ^b^	496.93	70.89	197.50	8.86
SN9714	20.14 ±1.01 ^b^	244.44	71.37	215.60	8.80
CJN6	19.29 ±1.30 ^b^	492.92	69.08	201.20	9.09
ZN106	20.80 ± 1.08 ^ab^	395.95	68.36	180.72	9.19
ZN65	21.06 ± 1.41 ^ab^	456.56	68.96	203.60	9.11
THN	21.39 ± 0.84 ^ab^	500.00	68.24	197.59	9.20
WGN1	21.24 ± 1.61 ^ab^	470.70	68.36	207.20	9.19
YN12	21.28 ± 1.08 ^ab^	542.00	69.92	203.61	8.98

Rc (%): relative crystallinity; Imax: maximum scattering intensity, S_max_: scattering vector at the lamellar peak maximum; ∆S: peak width; D: lamellar distance. Data are used with replications represented as means ± standard deviation. Different superscript letters within the same column indicate significant differences at *p* ≤ 0.05.

**Table 4:** Pasting properties of ten different waxy rice varieties.

Varieties	PV (cP)	TV (cP)	BDV (cP)	FV (cP)	SV (cP)	PT (min)	PT (°C)
NGXN	2261 ± 31 ^h^	1473 ± 21 ^d^	788 ± 13 ^i^	1714 ± 5 ^ef^	241 ± 11 ^g^	3.91 ± 0.03 ^e^	72.80 ± 0.10 ^d^
ZN19	2415 ± 31 ^g^	1458 ± 27 ^d^	957 ± 21 ^g^	1730 ± 9 ^e^	272 ± 8 ^de^	3.93 ± 0.04 ^e^	72.50 ± 0.21 ^de^
GMN2	2842 ± 36 ^c^	1795 ± 25 ^bc^	1047 ± 11 ^f^	2587 ± 6 ^a^	792 ± 11 ^a^	4.33 ± 0.04 ^a^	74.10 ± 0.15 ^b^
SN9714	2752 ± 46 ^d^	1345 ± 25 ^f^	1407 ± 13 ^a^	1607 ± 7 ^i^	262 ± 5 ^ef^	3.67 ± 0.01 ^h^	70.95 ± 0.04 ^g^
CJN6	2541 ± 39 ^ef^	1403 ± 25 ^e^	1138 ± 9 ^e^	1669 ± 11 ^g^	266 ± 7 ^e^	3.53 ± 0.02 ^i^	71.20 ± 0.15 ^g^
ZN106	3130 ± 16 ^b^	1833 ± 11 ^a^	1297 ± 6 ^d^	2165 ± 11 ^b^	332 ± 6 ^b^	4.07 ± 0.02 ^c^	71.55 ± 0.33 ^f^
ZN65	2643 ± 34 ^e^	1310 ± 15 ^g^	1333 ± 14 ^c^	1635 ± 10 ^h^	325 ± 6 ^bc^	3.73 ± 0.01 ^g^	71.70 ± 0.21 ^f^
THN	2134 ± 26 ^i^	1171 ± 14 ^h^	963 ± 7 ^h^	1375 ± 7 ^j^	204 ± 3 ^h^	3.80 ± 0.02 ^f^	72.45 ± 0.20 ^e^
WGN1	3205 ± 26 ^a^	1826 ± 9 ^ab^	1379 ± 19 ^b^	2138 ± 11 ^c^	312 ± 14 ^c^	4.00 ± 0.03 ^d^	73.35 ± 0.15 ^c^
YN12	2519 ± 29 ^ef^	1771 ± 16 ^c^	748 ± 10 ^j^	2053 ± 7 ^d^	282 ± 6 ^d^	4.27 ± 0.02 ^b^	74.90 ± 0.08 ^a^

PV: peak viscosity; TV: trough viscosity; BD: breakdown viscosity; FV: final viscosity; SV: setback viscosity; PT (min): pasting time; PT (°C): pasting temperature. Data are used with replications represented as means ± standard deviation. Different superscript letters within the same column indicate significant differences at *p* ≤ 0.05.

**Table 5:** Gelatinization and retrogradation of ten different waxy rice varieties.

Varieties	Gelatinization				Retrogradation			
	T_o_ (°C)	T_p_ (°C)	T_c_ (°C)	ΔH (J/g)	T_o_ (°C)	T_p_ (°C)	T_c_ (°C)	ΔH (J/g)
NGXN	62.8 ± 1.6 ^d^	70.3 ± 0.3 ^d^	78.4 ± 0.4 ^cd^	14.8 ± 0.3 ^a^	63.4 ± 0.4 ^b^	65.1 ± 0.3 ^b^	69.0 ± 0.5 ^a^	0.2 ± 0.0 ^d^
ZN19	64.7 ± 0.7 ^bc^	71.1 ± 0.4 ^c^	78.9 ± 0.5 ^bc^	12.7 ± 0.2 ^d^	65.1 ± 0.3 ^a^	66.9 ± 0.4 ^a^	69.2 ± 0.3 ^a^	0.2 ± 0.0 ^b^
GMN2	65.7 ± 0.5 ^ab^	72.2 ± 0.3 ^a^	79.4 ± 0.4 ^b^	12.6 ± 0.2 ^de^	47.8 ± 0.3 ^f^	57.0 ± 0.5 ^d^	59.0 ± 0.3 ^b^	0.3 ± 0.0 ^a^
SN9714	62.7 ± 0.6 ^d^	70.1 ± 0.4 ^d^	77.9 ± 0.3 ^de^	12.3 ± 0.2 ^e^	57.7 ± 0.4 ^c^	56.1 ± 0.3 ^e^	57.6 ± 0.4 ^de^	0.1 ± 0.0 ^f^
CJN6	60.2 ± 0.0 ^e^	68.2 ± 0.3 e	77.4 ± 0.3 ^e^	12.8 ± 0.2 ^d^	46.9 ± 0.7 ^g^	49.8 ± 0.4 ^h^	57.0 ± 0.4 ^e^	0.1 ± 0.0 ^i^
ZN106	63.2 ± 0.8 ^d^	69.9 ± 0.3 ^d^	77.4 ± 0.4 ^e^	12.6 ± 0.2 ^de^	52.9 ± 0.6 ^d^	54.6 ± 0.5 ^f^	57.4 ± 0.4 ^e^	0.1 ± 0.0 ^g^
ZN65	63.5 ± 0.5 ^cd^	71.6 ± 0.3 ^bc^	79.4 ± 0.3 ^b^	13.0 ± 0.2 ^d^	64.4 ± 0.6 ^a^	67.3 ± 0.5 ^a^	68.9 ± 0.2 ^a^	0.1 ± 0.0 ^h^
THN	65.3 ± 0.6 ^ab^	72.0 ± 0.2 ^ab^	79.1 ± 0.2 ^b^	13.9 ± 0.2 ^b^	50.0 ± 0.6 ^e^	52.6 ± 0.5 ^g^	59.1 ± 0.3 ^b^	0.1 ± 0.0 ^e^
WGN1	65.6 ± 0.5 ^ab^	71.6 ± 0.4 ^bc^	79.8 ± 0.3 ^b^	13.4 ± 0.3 ^c^	52.2 ± 0.5 ^d^	56.7 ± 0.4 ^de^	58.2 ± 0.3 ^cd^	0.2 ± 0.0 ^d^
YN12	66.4 ± 0.4 ^a^	72.0 ± 0.2 ^ab^	80.8 ± 0.5 ^a^	13.5 ± 0.2 ^c^	49.4 ± 0.3 ^e^	57.7 ± 0.4 ^c^	58.7 ± 0.4 ^bc^	0.2 ± 0.0 ^c^

T_o_: onset temperature; T_p_: peak temperature; T_c_: conclusion temperature; ΔH: gelatinization enthalpy. Data are used with replications represented as means ± standard deviation. Different superscript letters within the same column indicate significant differences at *p* ≤ 0.05.

**Table 6:** Digestion kinetics of ten different waxy rice starches.

Samples	Log	
	*k* 10^−2^ (min^−1^)	*C_res_* (%)
NGXN	1.54 ± 0.08 ^d^	5.63 ± 0.11 ^d^
ZN19	1.71 ± 0.04 ^c^	9.06 ± 0.04 ^b^
GMN2	1.47 ± 0.03 ^e^	4.58 ± 0.03 ^f^
SN9714	1.74 ± 0.03 ^bc^	3.97 ± 0.03 ^i^
CJN6	1.79 ± 0.02 ^b^	5.20 ± 0.02 ^e^
ZN106	1.90 ± 0.02 ^a^	8.66 ± 0.02 ^c^
ZN65	1.72 ± 0.02 ^c^	4.38 ± 0.02 ^h^
THN	1.60 ± 0.02 ^d^	4.49 ± 0.03 ^g^
WGN1	1.55 ± 0.03 ^d^	1.16 ± 0.02 ^j^
YN12	1.48 ± 0.02 ^e^	9.81 ± 0.03 ^a^

*k*: rate coefficient; *C_res_*: undigested starch at infinite. Data are used with replications represented as means ± standard deviation. Different superscript letters within the same column indicate significant differences at *p* ≤ 0.05.

## Data Availability

The original contributions presented in this study are included in the article/Supplementary Materials. Further inquiries can be directed to the corresponding author.

## Supplementary Materials

The following supporting information can be downloaded at https://www.mdpi.com/article/10.3390/foods14234130/s1.
