# Supplementary figures and images for: Investigating the Impact of Amylopectin Chain-Length Distribution on the Structural and Functional Properties of Waxy Rice Starch
Source: Foods. 2025 Dec 2;14(23):4130. doi: 10.3390/foods14234130 (PMC12692052; doi:10.3390/foods14234130)

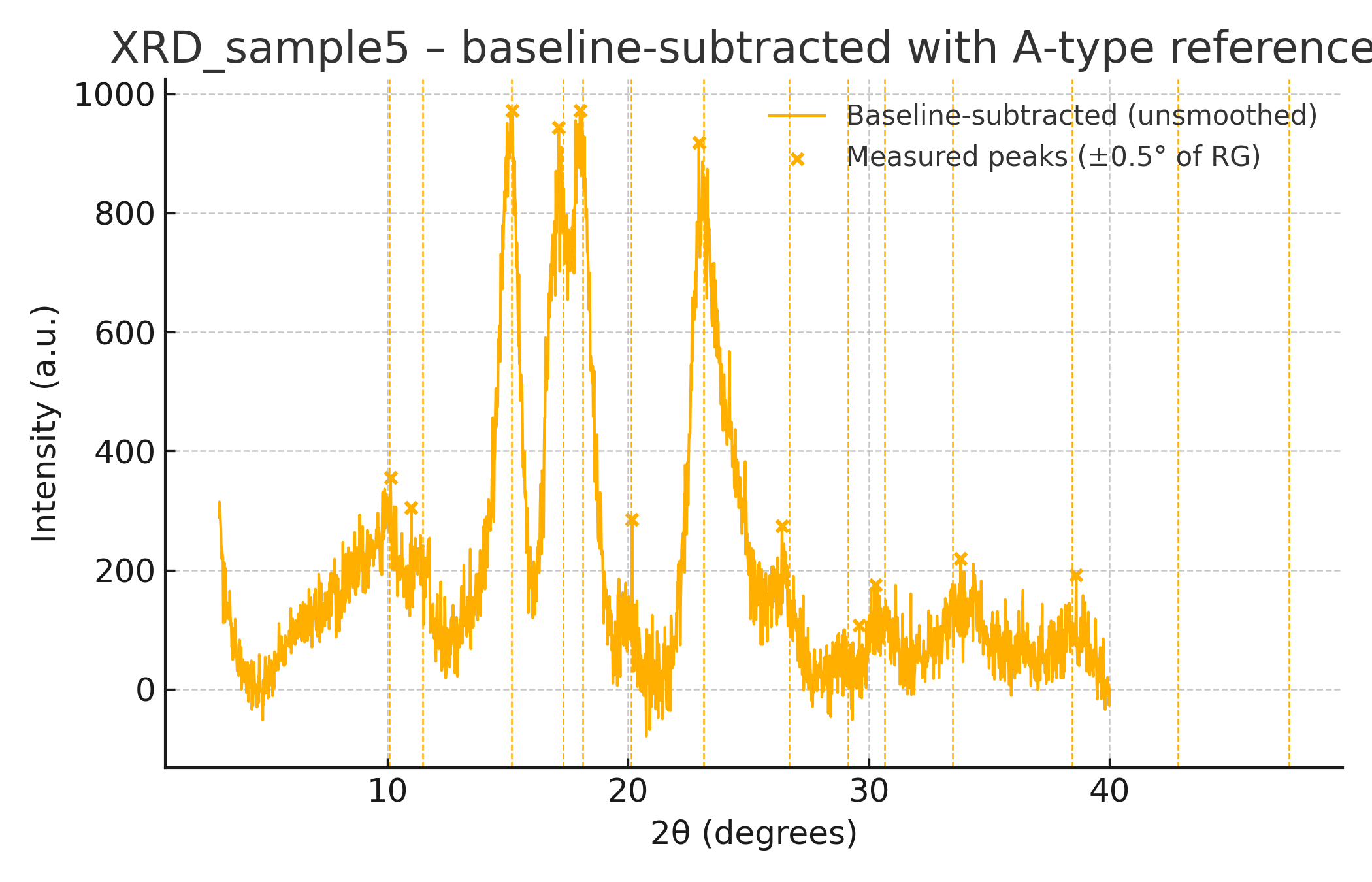

Supplement: Supplementary file 1 [file foods-14-04130-s001.zip › Supplementary data (XRD Analysed data)/Supplementary data (XRD Analysed data)/CJN6/XRD_sample5_corrected_peaks.png]

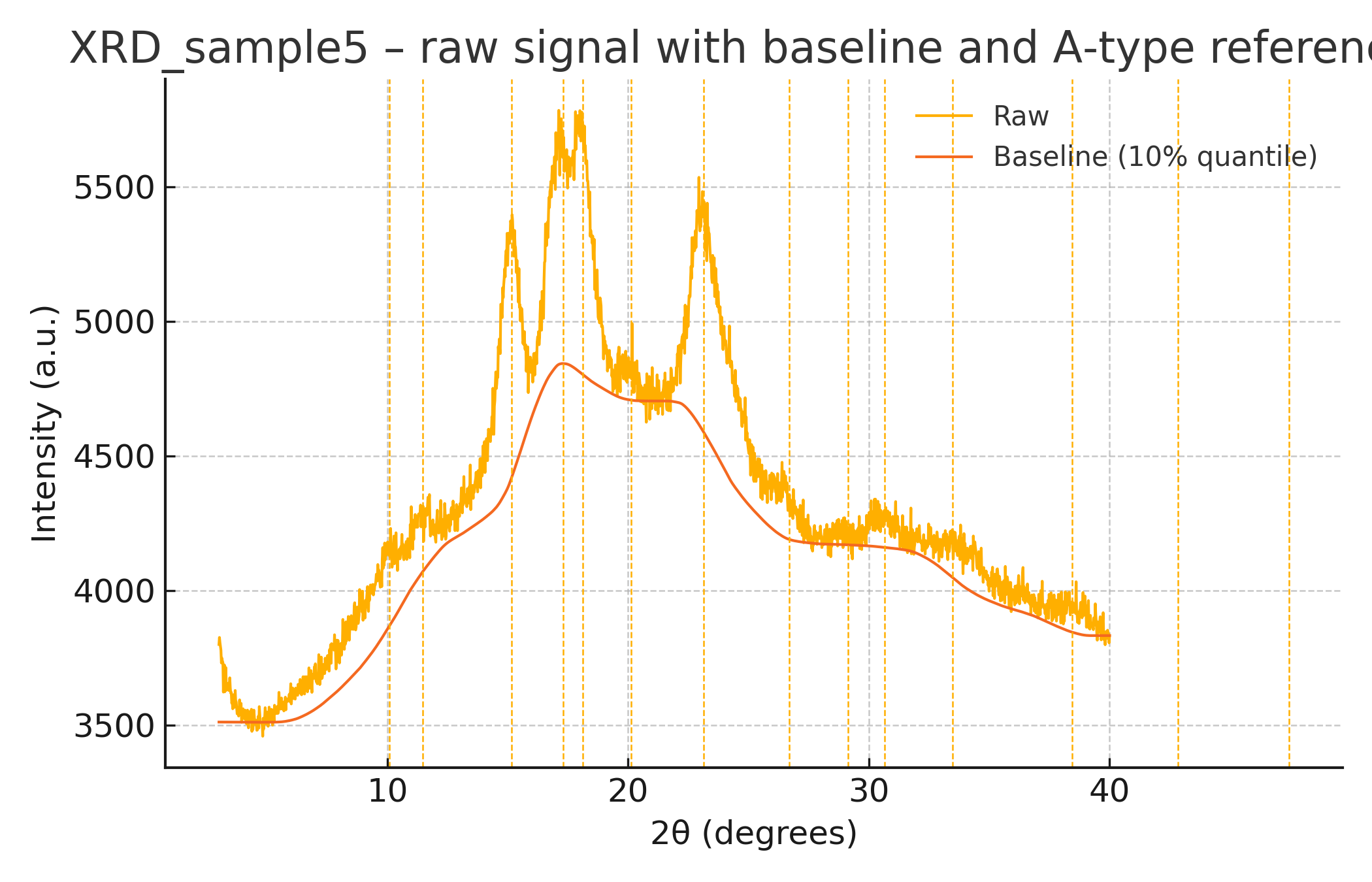

Supplement: Supplementary file 1 [file foods-14-04130-s001.zip › Supplementary data (XRD Analysed data)/Supplementary data (XRD Analysed data)/CJN6/XRD_sample5_raw_baseline.png]

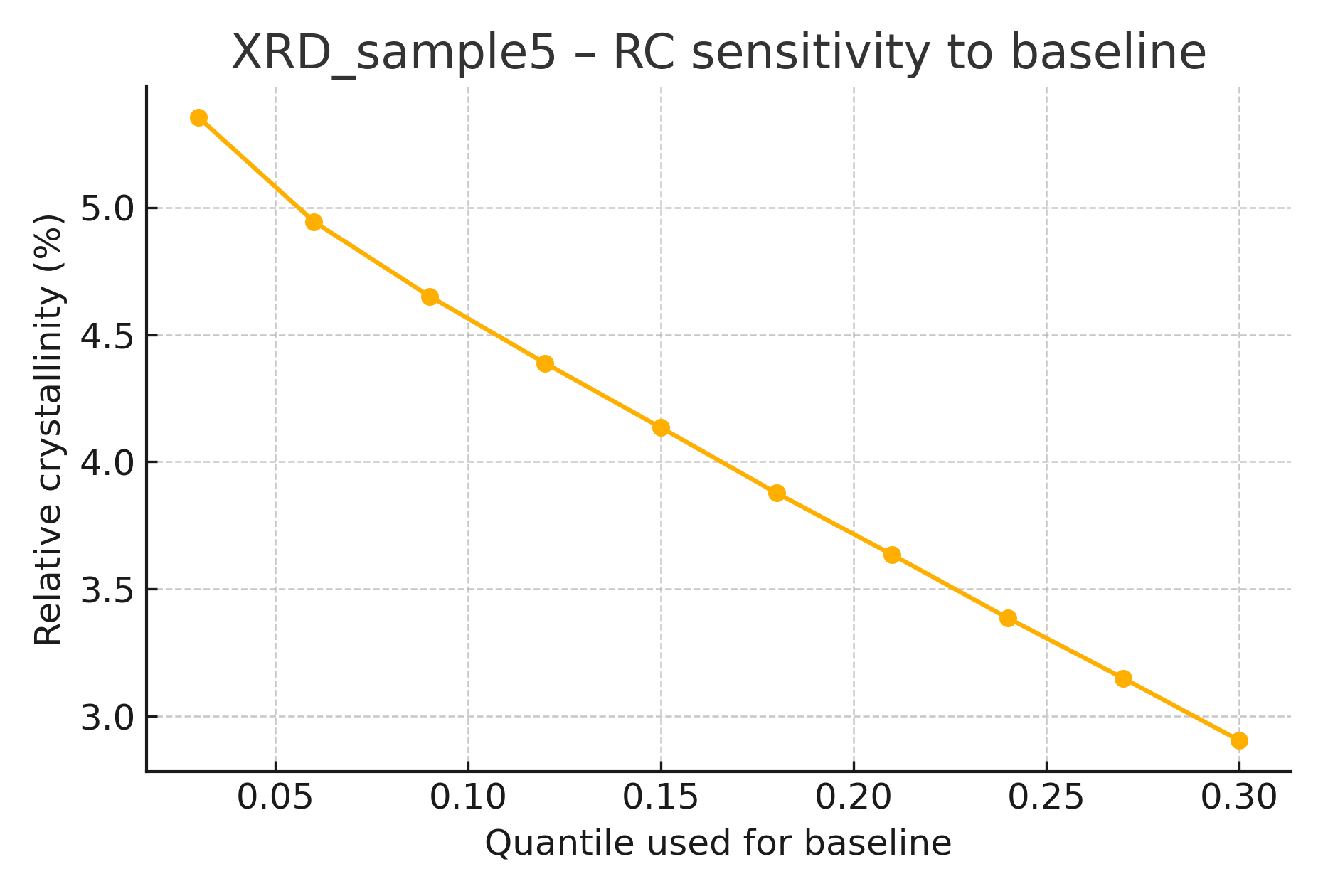

Supplement: Supplementary file 1 [file foods-14-04130-s001.zip › Supplementary data (XRD Analysed data)/Supplementary data (XRD Analysed data)/CJN6/XRD_sample5_RC_sensitivity.png]

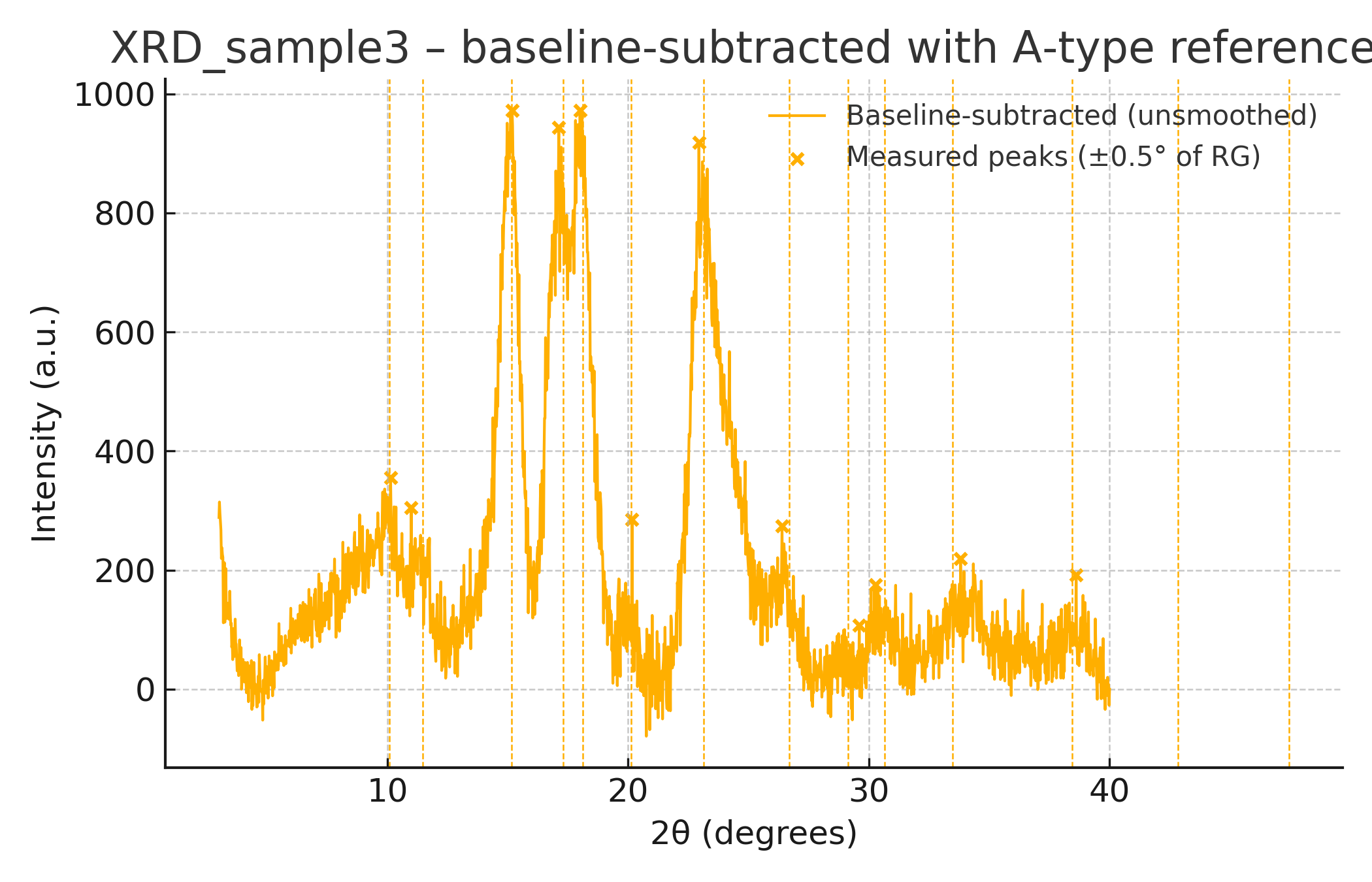

Supplement: Supplementary file 1 [file foods-14-04130-s001.zip › Supplementary data (XRD Analysed data)/Supplementary data (XRD Analysed data)/GMN2/XRD_sample3_corrected_peaks.png]

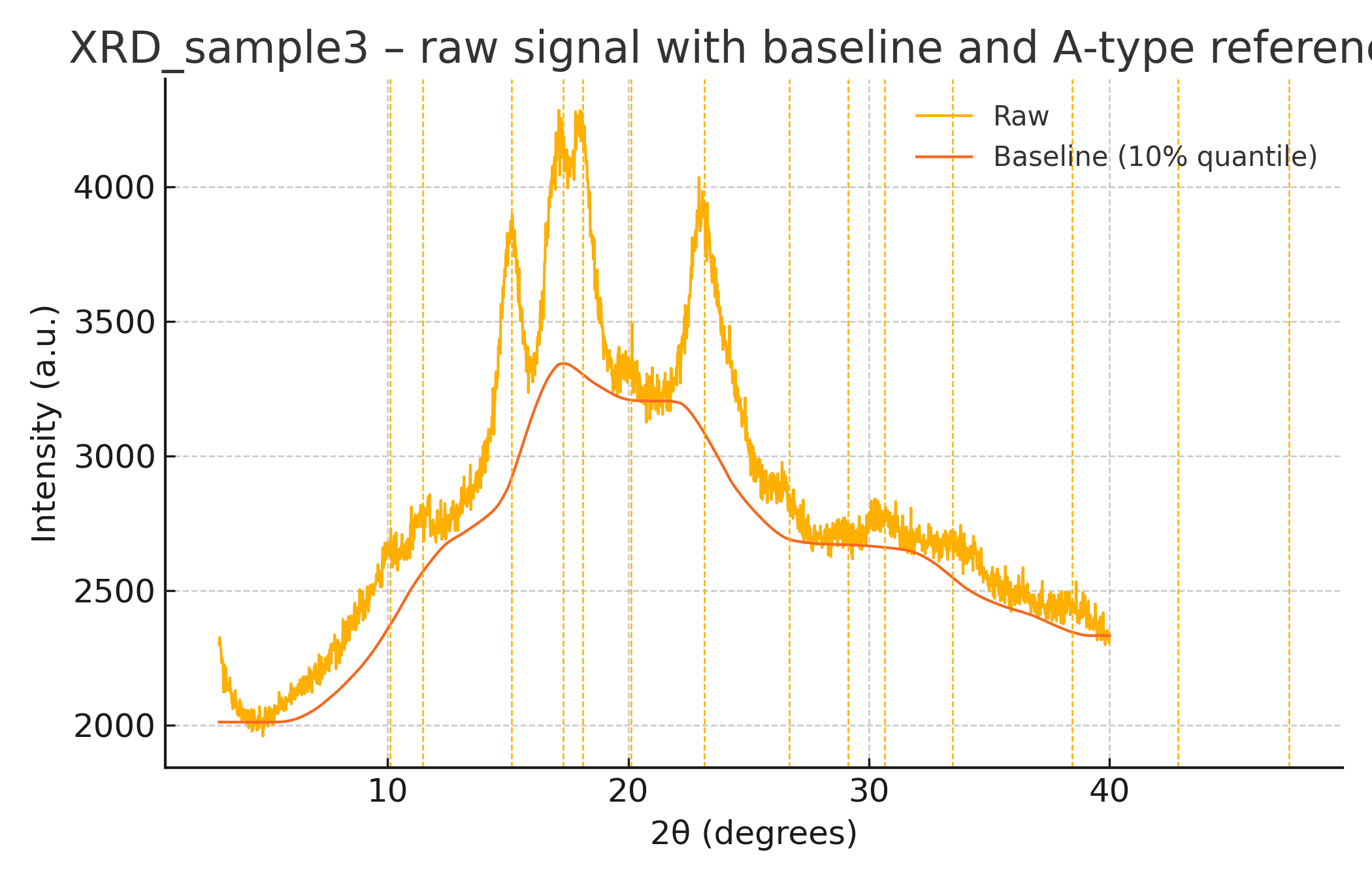

Supplement: Supplementary file 1 [file foods-14-04130-s001.zip › Supplementary data (XRD Analysed data)/Supplementary data (XRD Analysed data)/GMN2/XRD_sample3_raw_baseline.png]

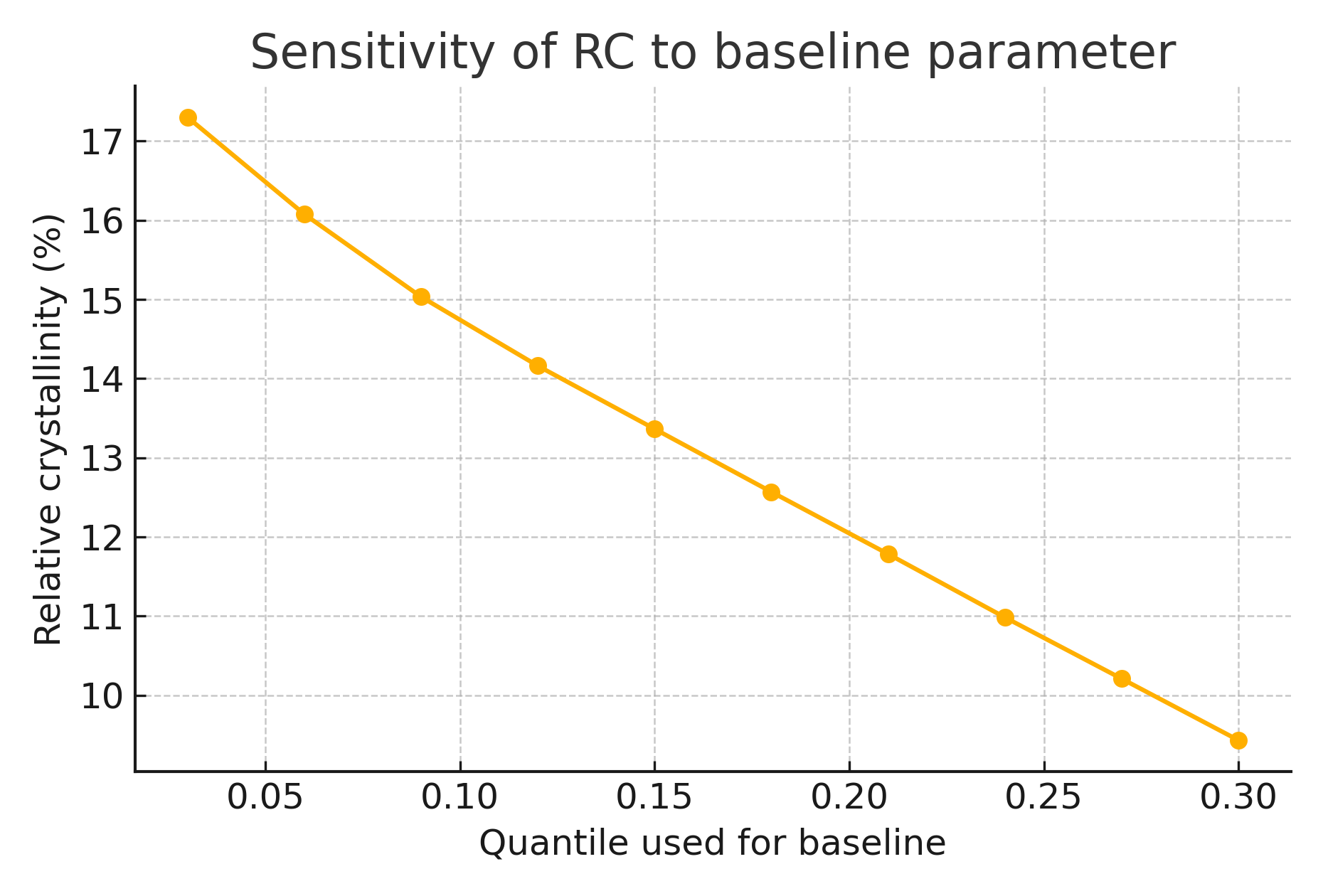

Supplement: Supplementary file 1 [file foods-14-04130-s001.zip › Supplementary data (XRD Analysed data)/Supplementary data (XRD Analysed data)/NGXN/Fig_RC_sensitivity.png]

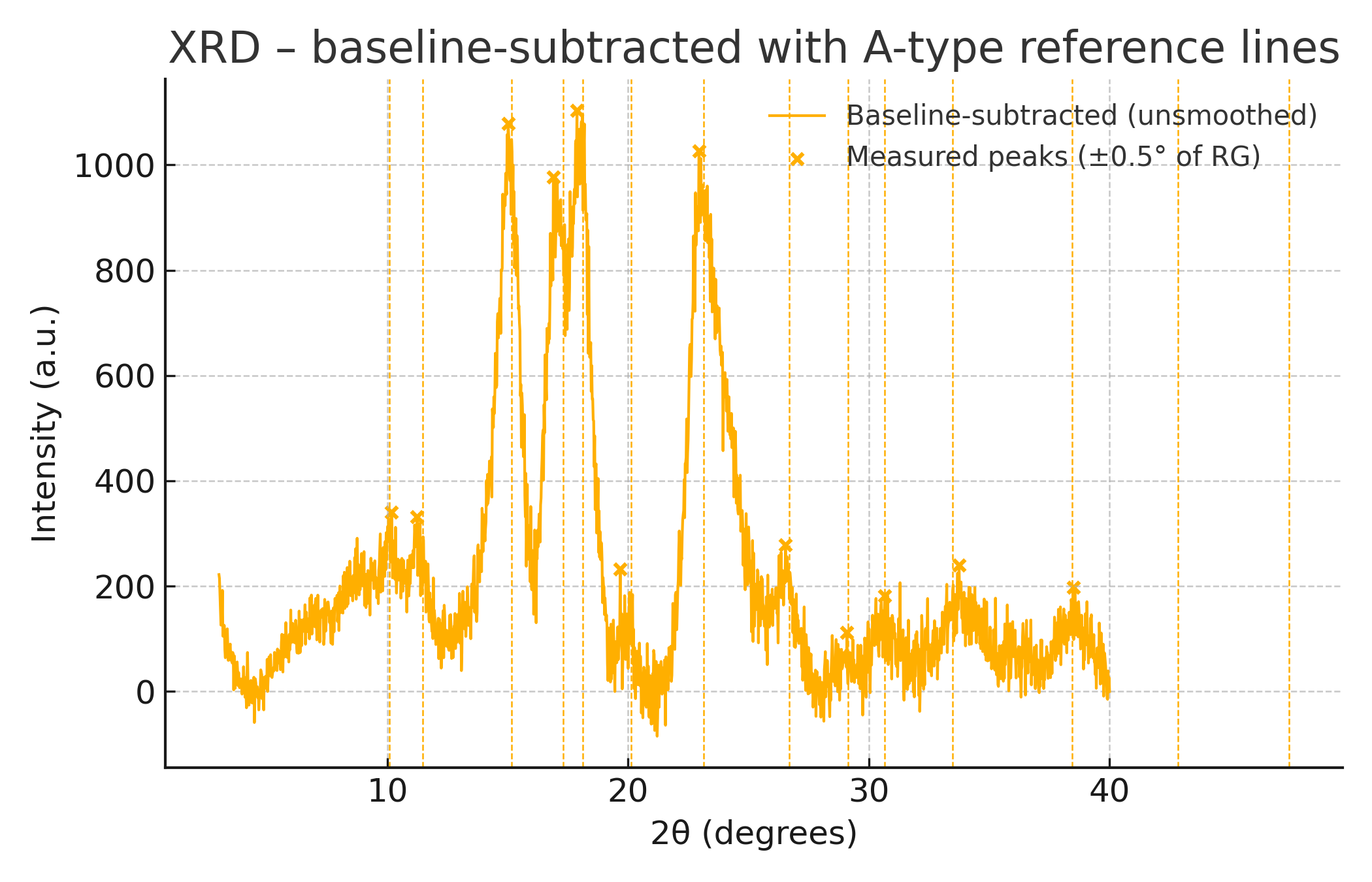

Supplement: Supplementary file 1 [file foods-14-04130-s001.zip › Supplementary data (XRD Analysed data)/Supplementary data (XRD Analysed data)/NGXN/Fig_XRD_corrected_with_lines.png]

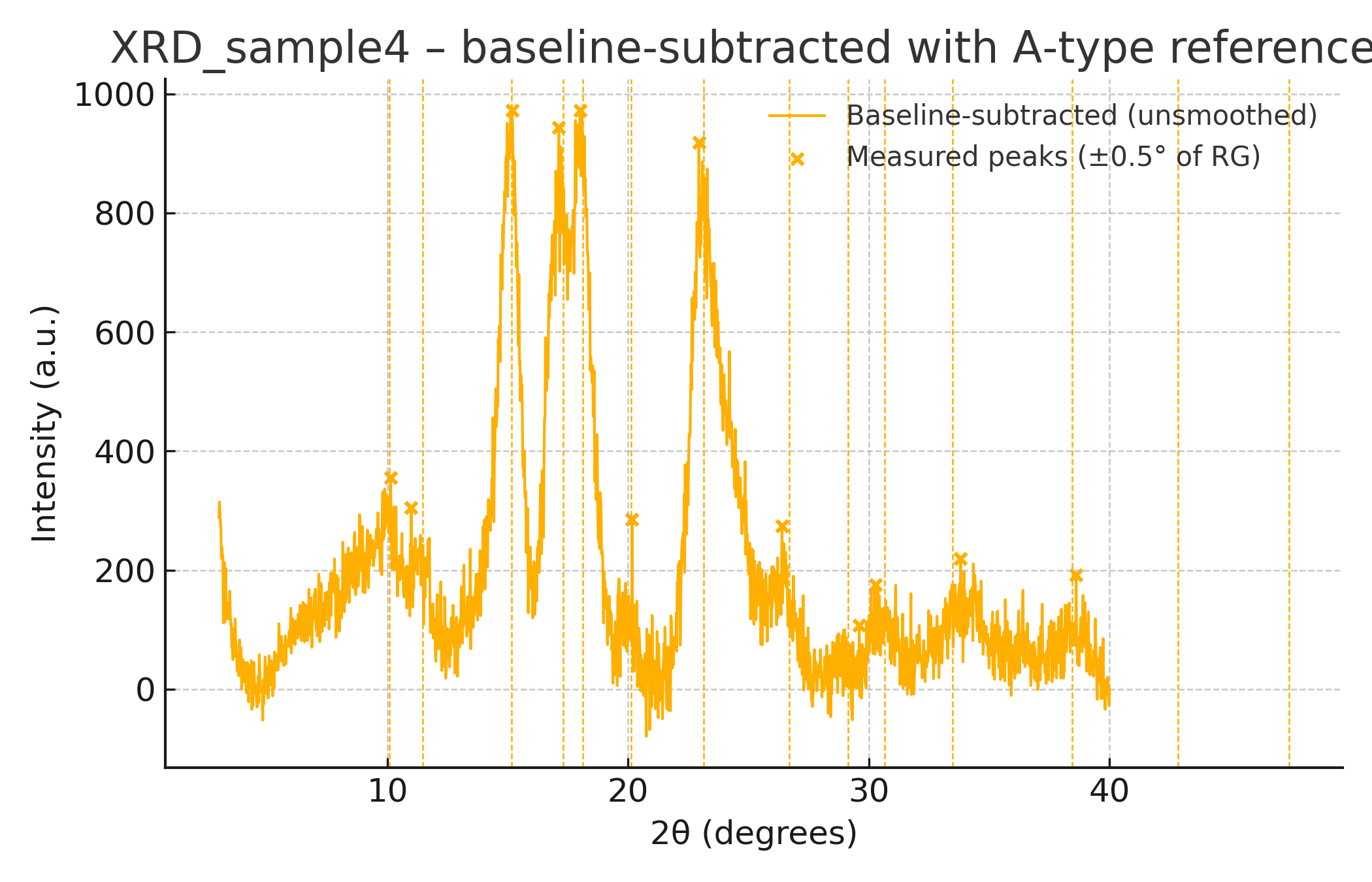

Supplement: Supplementary file 1 [file foods-14-04130-s001.zip › Supplementary data (XRD Analysed data)/Supplementary data (XRD Analysed data)/SN9714/XRD_sample4_corrected_peaks.png]

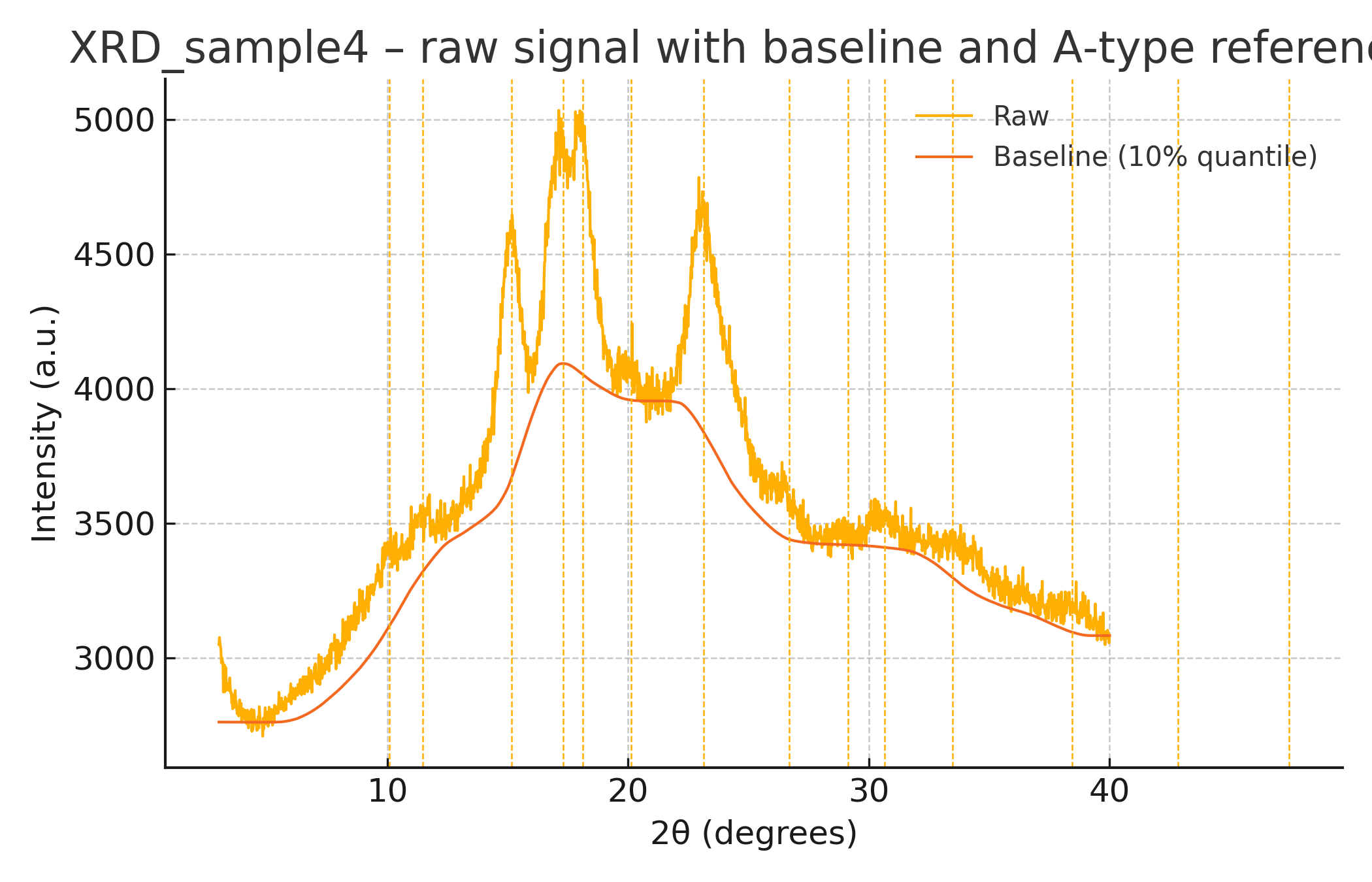

Supplement: Supplementary file 1 [file foods-14-04130-s001.zip › Supplementary data (XRD Analysed data)/Supplementary data (XRD Analysed data)/SN9714/XRD_sample4_raw_baseline.png]

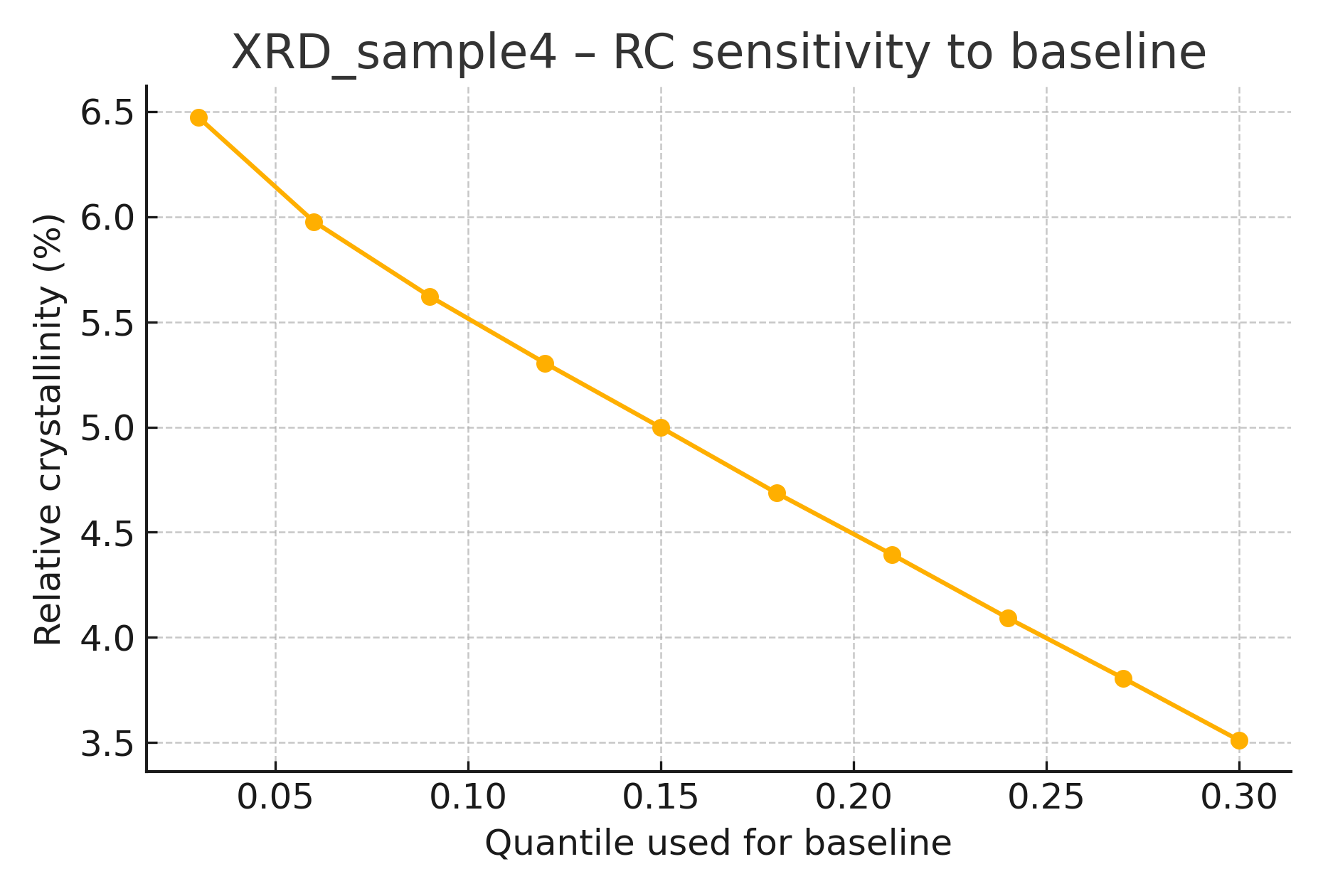

Supplement: Supplementary file 1 [file foods-14-04130-s001.zip › Supplementary data (XRD Analysed data)/Supplementary data (XRD Analysed data)/SN9714/XRD_sample4_RC_sensitivity.png]

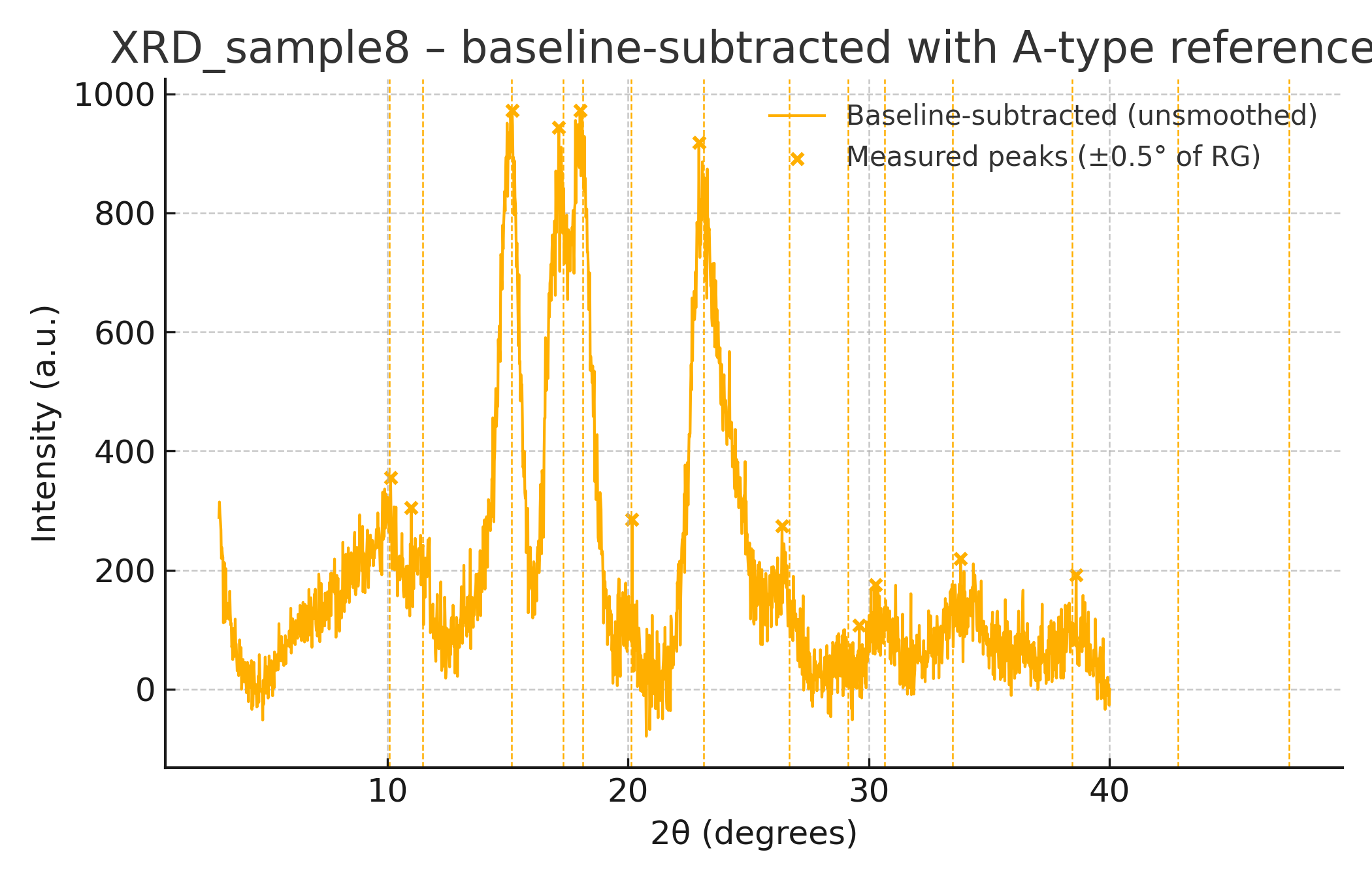

Supplement: Supplementary file 1 [file foods-14-04130-s001.zip › Supplementary data (XRD Analysed data)/Supplementary data (XRD Analysed data)/THN/XRD_sample8_corrected_peaks.png]

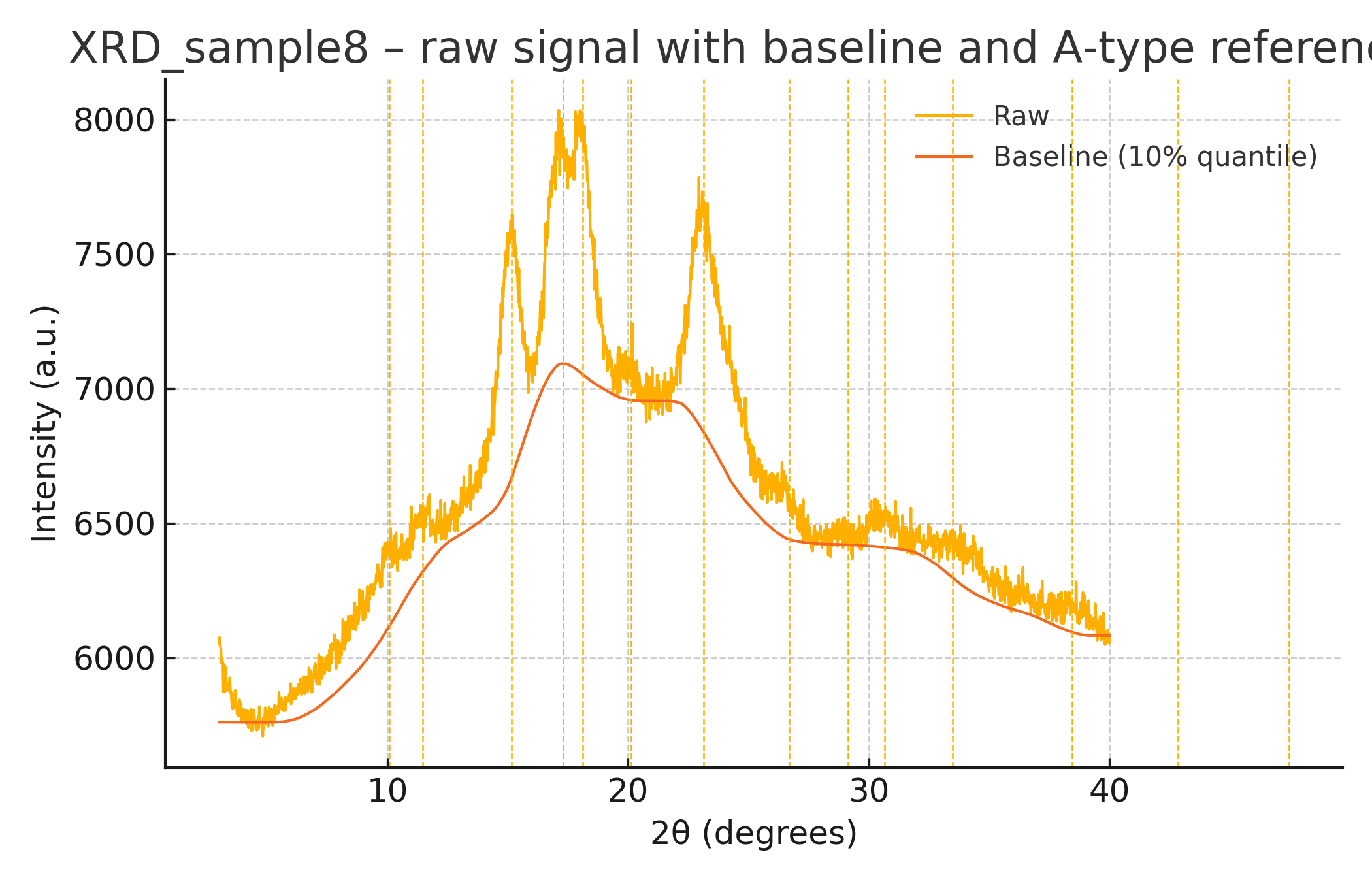

Supplement: Supplementary file 1 [file foods-14-04130-s001.zip › Supplementary data (XRD Analysed data)/Supplementary data (XRD Analysed data)/THN/XRD_sample8_raw_baseline.png]

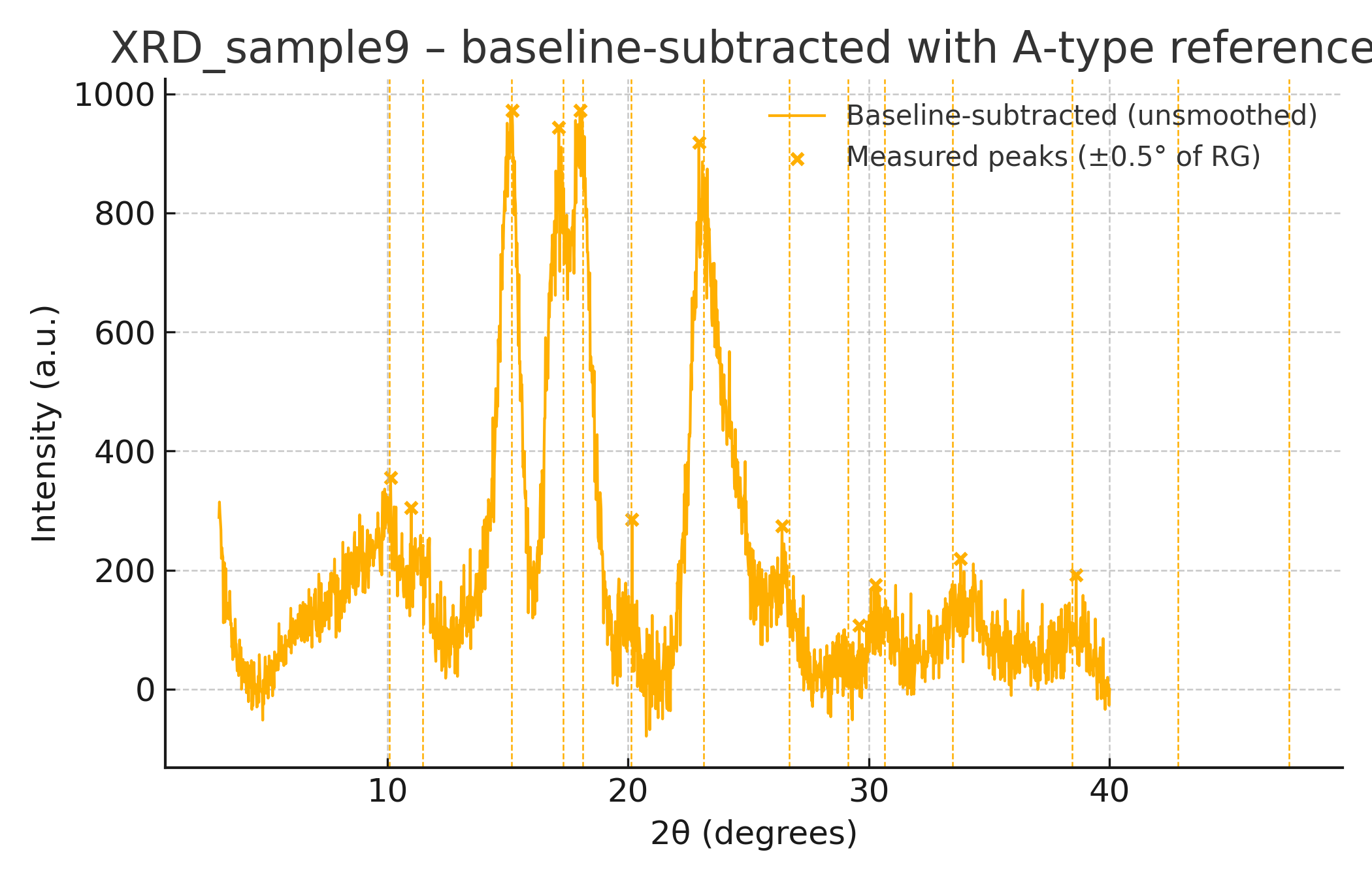

Supplement: Supplementary file 1 [file foods-14-04130-s001.zip › Supplementary data (XRD Analysed data)/Supplementary data (XRD Analysed data)/WGN1/XRD_sample9_corrected_peaks.png]

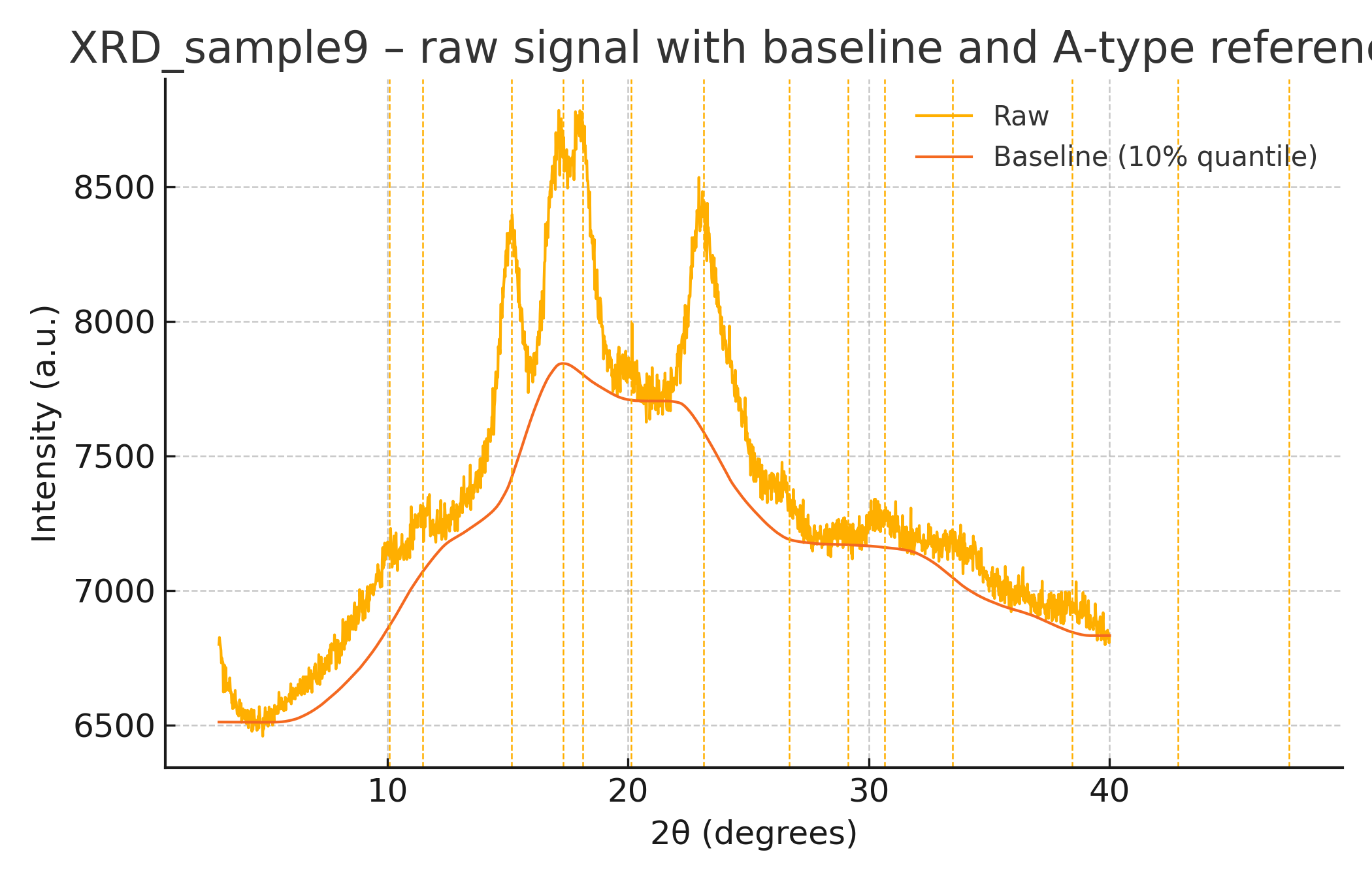

Supplement: Supplementary file 1 [file foods-14-04130-s001.zip › Supplementary data (XRD Analysed data)/Supplementary data (XRD Analysed data)/WGN1/XRD_sample9_raw_baseline.png]

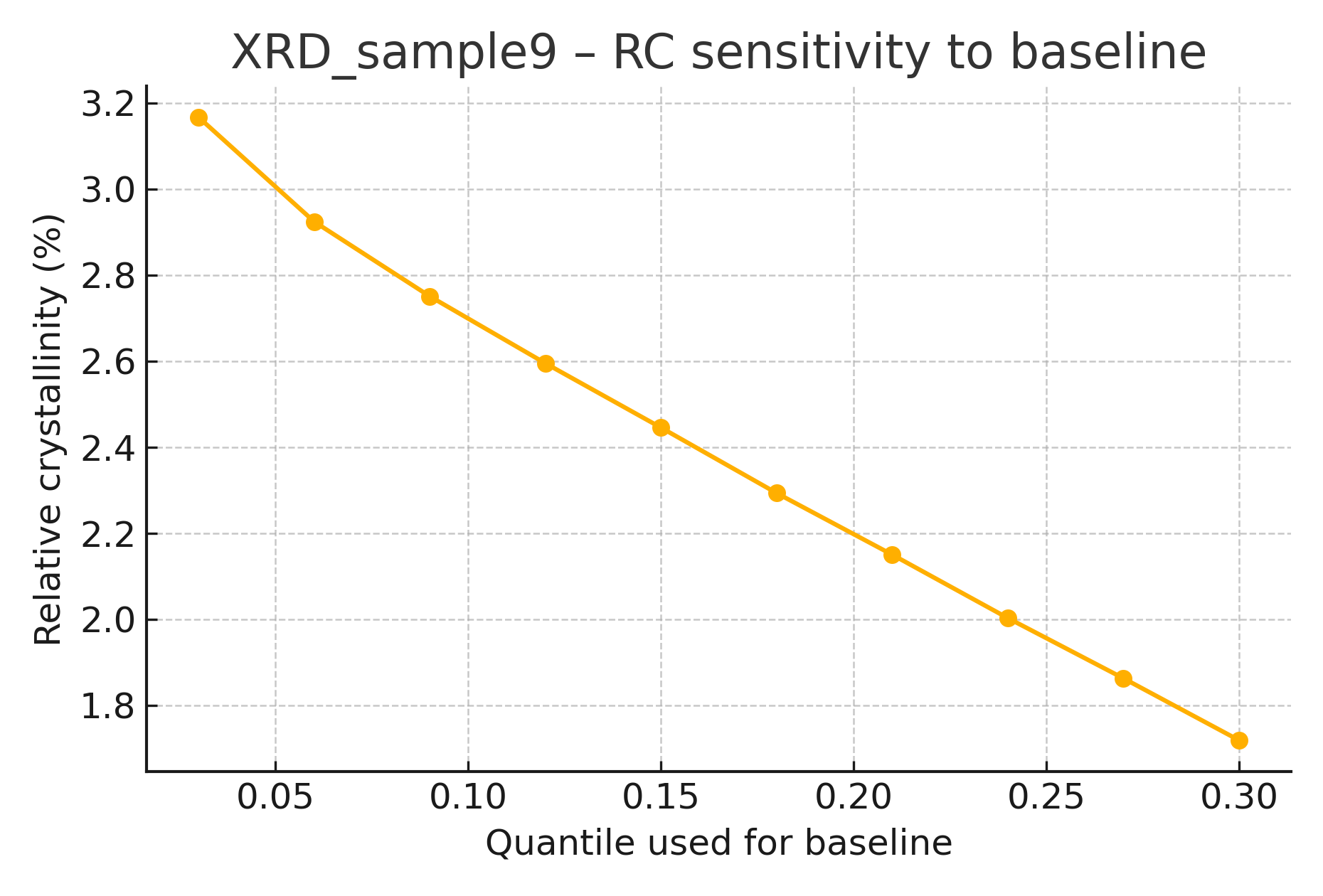

Supplement: Supplementary file 1 [file foods-14-04130-s001.zip › Supplementary data (XRD Analysed data)/Supplementary data (XRD Analysed data)/WGN1/XRD_sample9_RC_sensitivity.png]

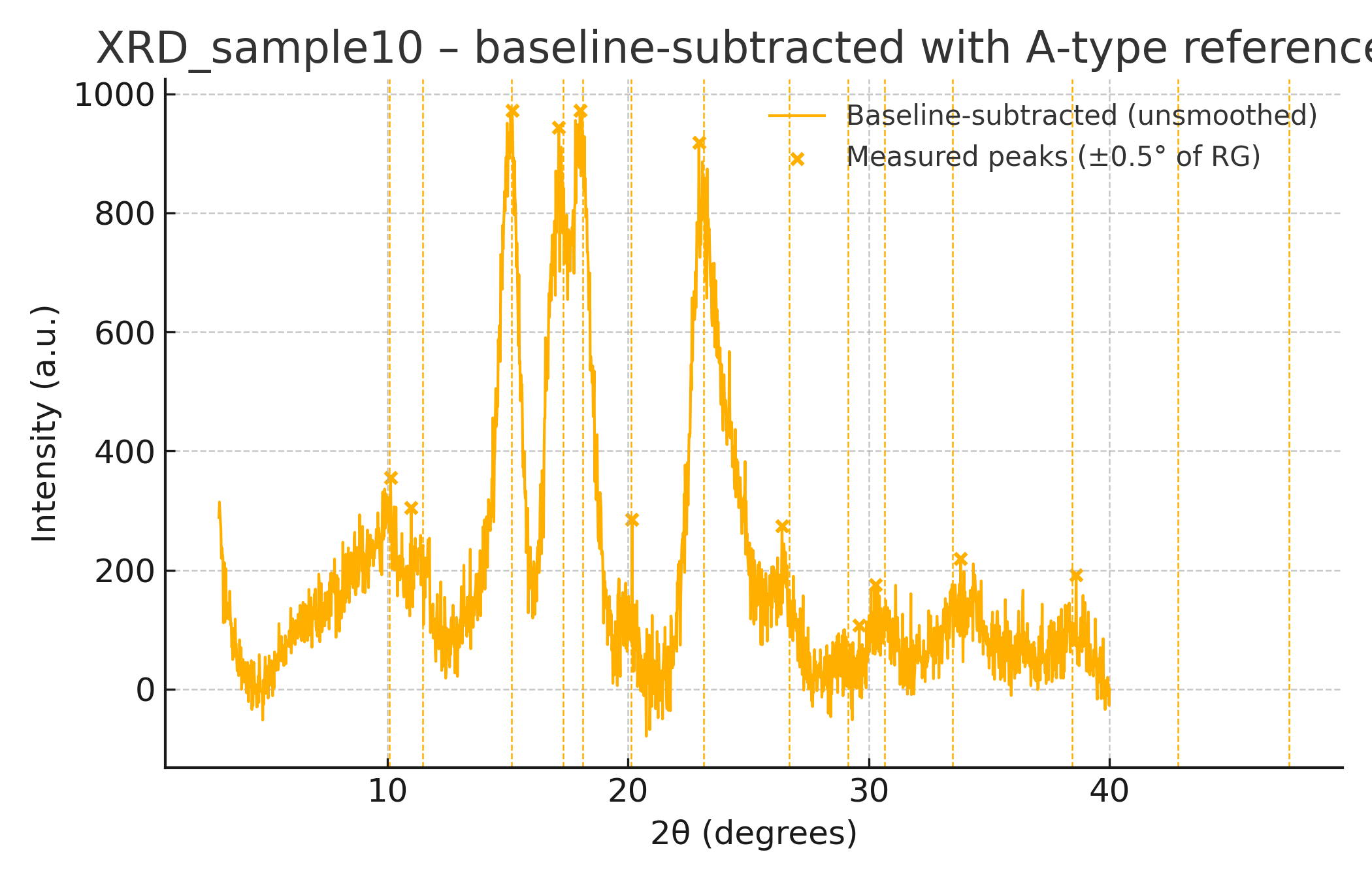

Supplement: Supplementary file 1 [file foods-14-04130-s001.zip › Supplementary data (XRD Analysed data)/Supplementary data (XRD Analysed data)/YN12/XRD_sample10_corrected_peaks.png]

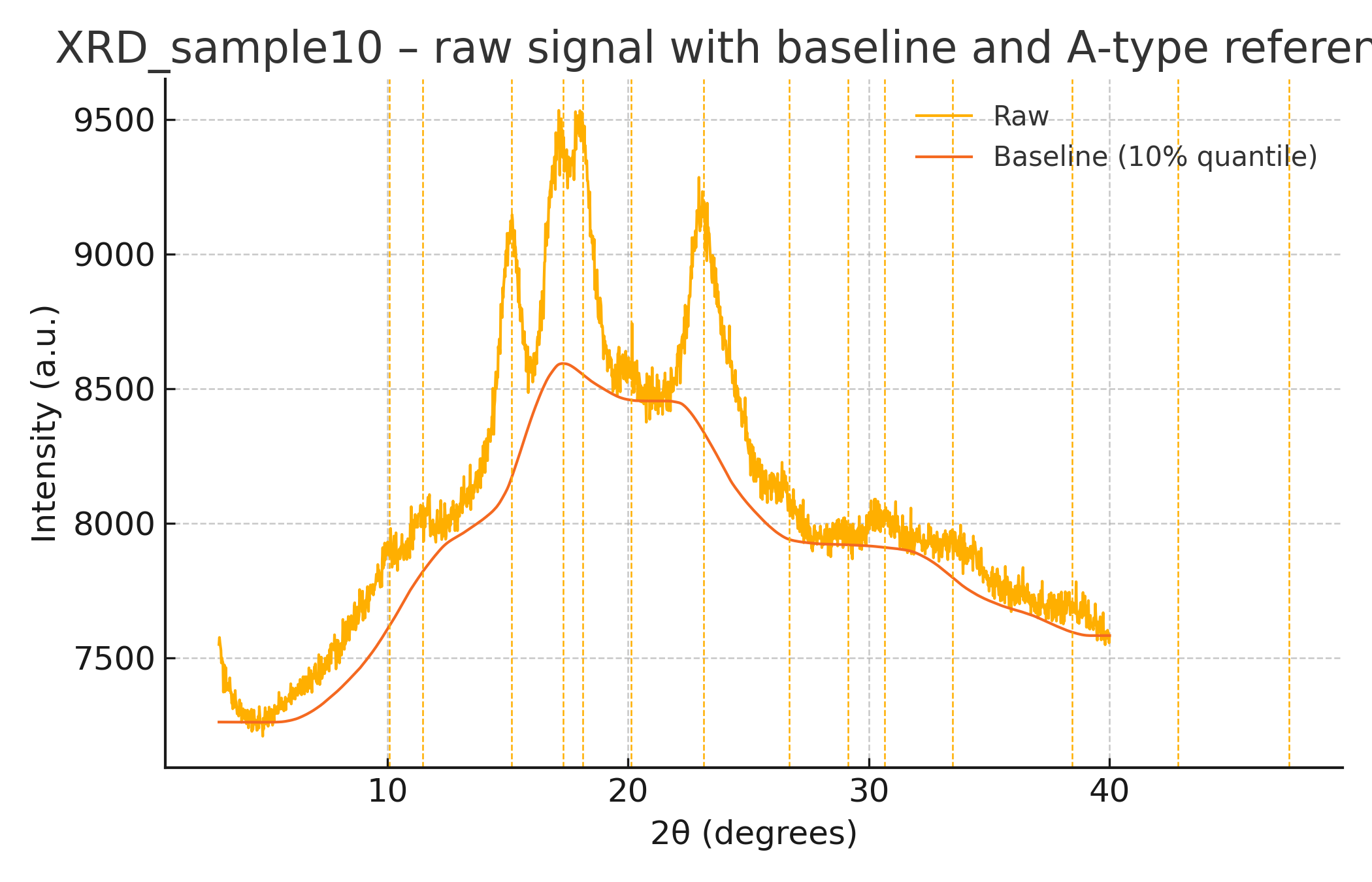

Supplement: Supplementary file 1 [file foods-14-04130-s001.zip › Supplementary data (XRD Analysed data)/Supplementary data (XRD Analysed data)/YN12/XRD_sample10_raw_baseline.png]

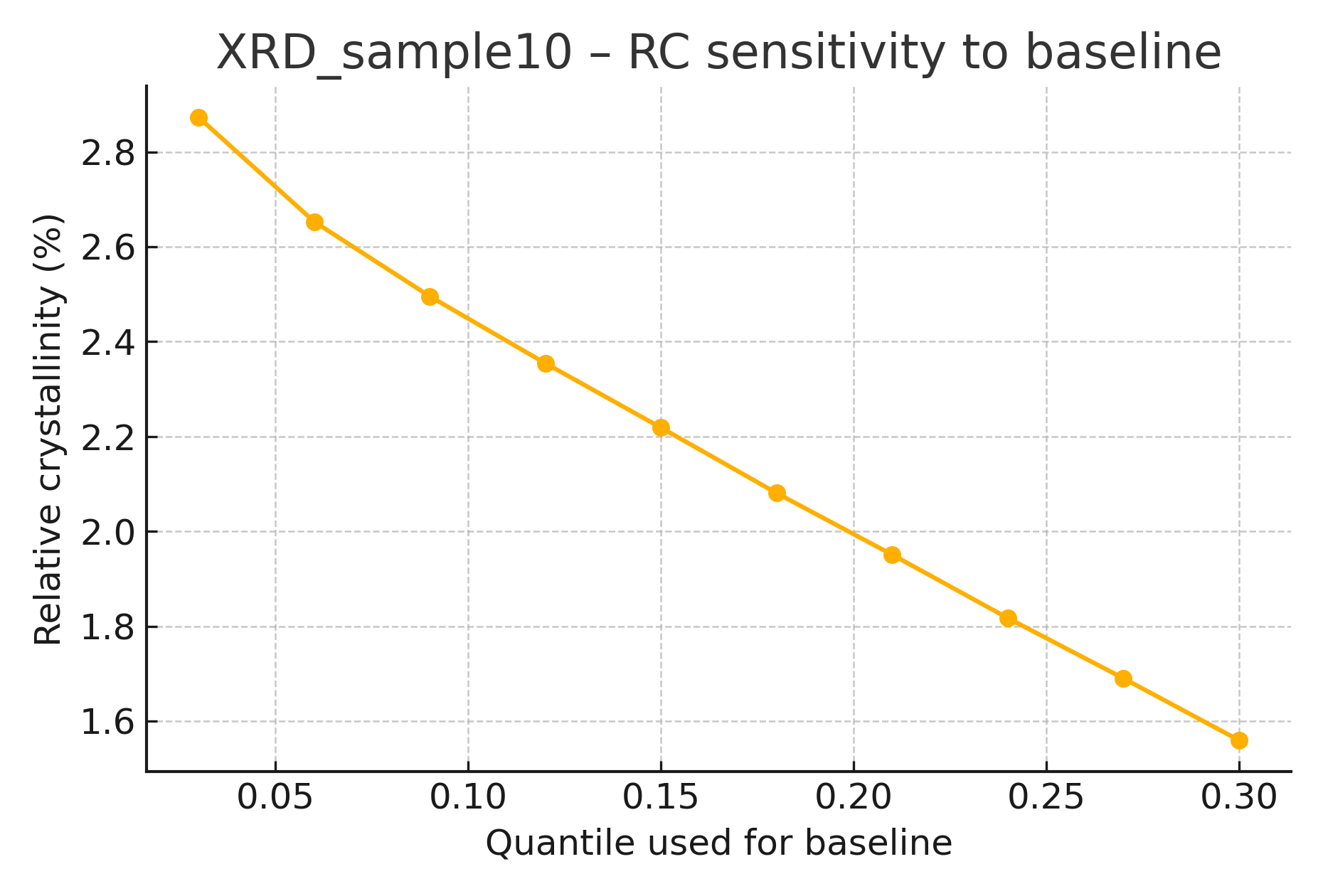

Supplement: Supplementary file 1 [file foods-14-04130-s001.zip › Supplementary data (XRD Analysed data)/Supplementary data (XRD Analysed data)/YN12/XRD_sample10_RC_sensitivity.png]

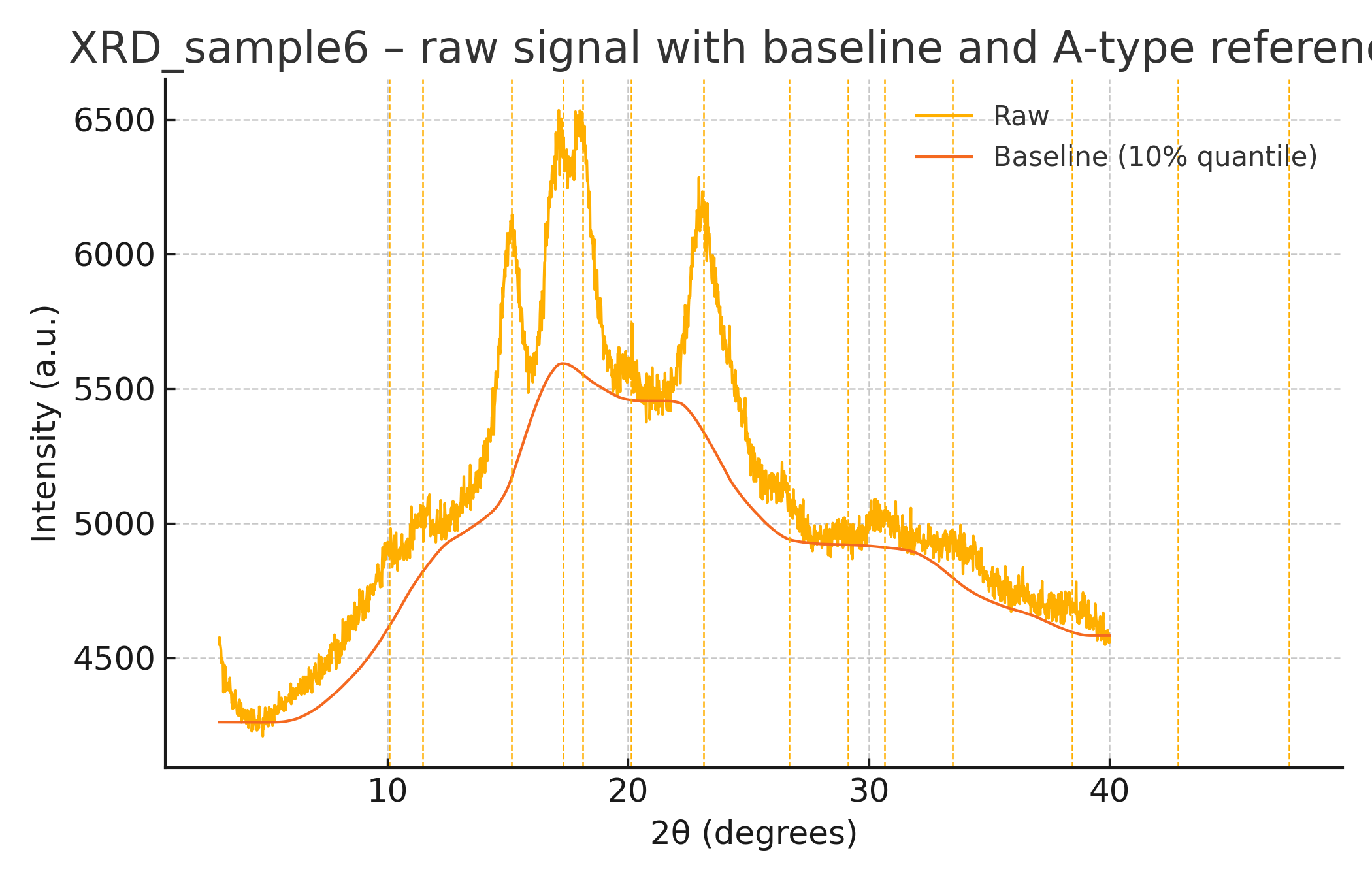

Supplement: Supplementary file 1 [file foods-14-04130-s001.zip › Supplementary data (XRD Analysed data)/Supplementary data (XRD Analysed data)/ZN106/XRD_sample6_raw_baseline.png]

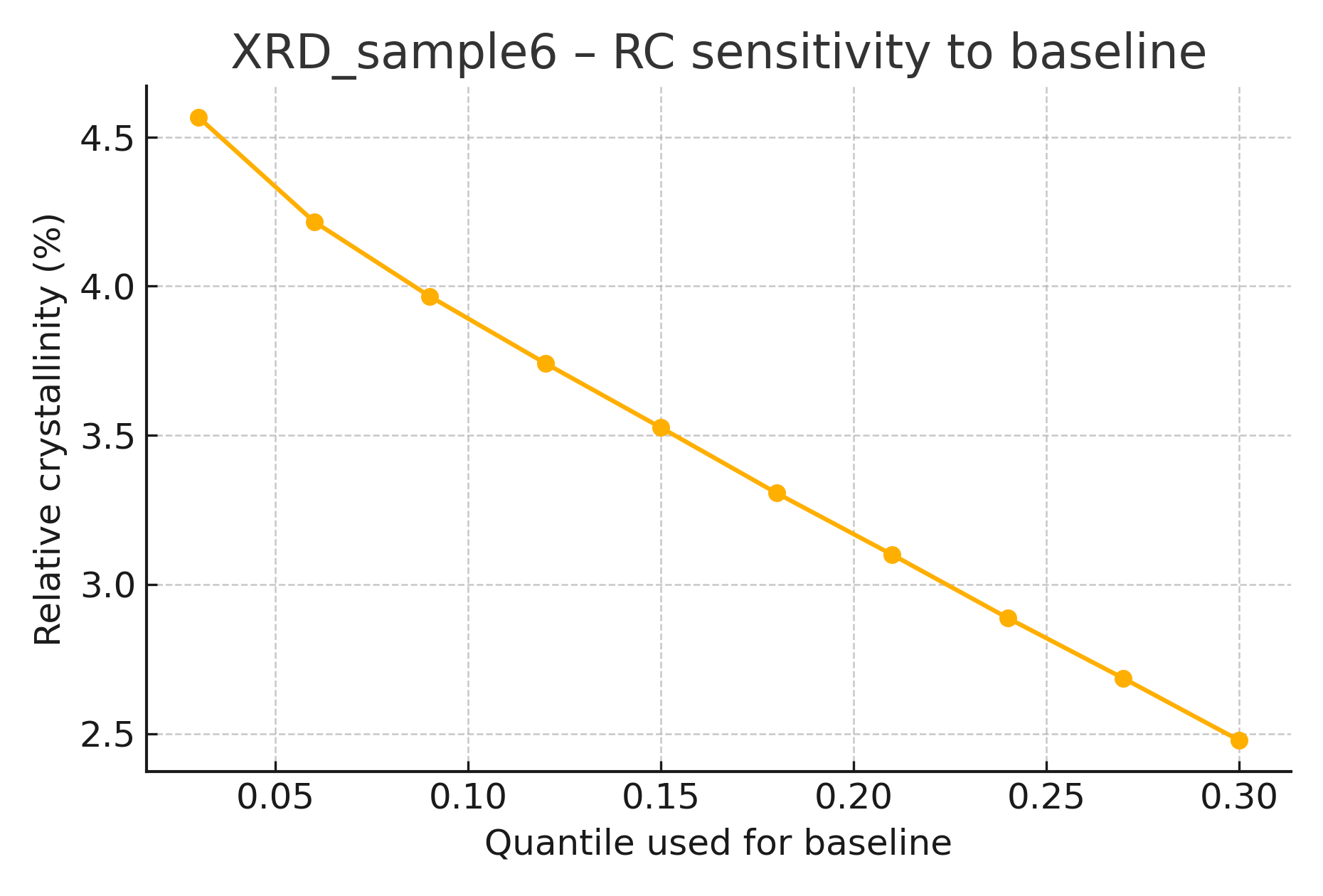

Supplement: Supplementary file 1 [file foods-14-04130-s001.zip › Supplementary data (XRD Analysed data)/Supplementary data (XRD Analysed data)/ZN106/XRD_sample6_RC_sensitivity.png]

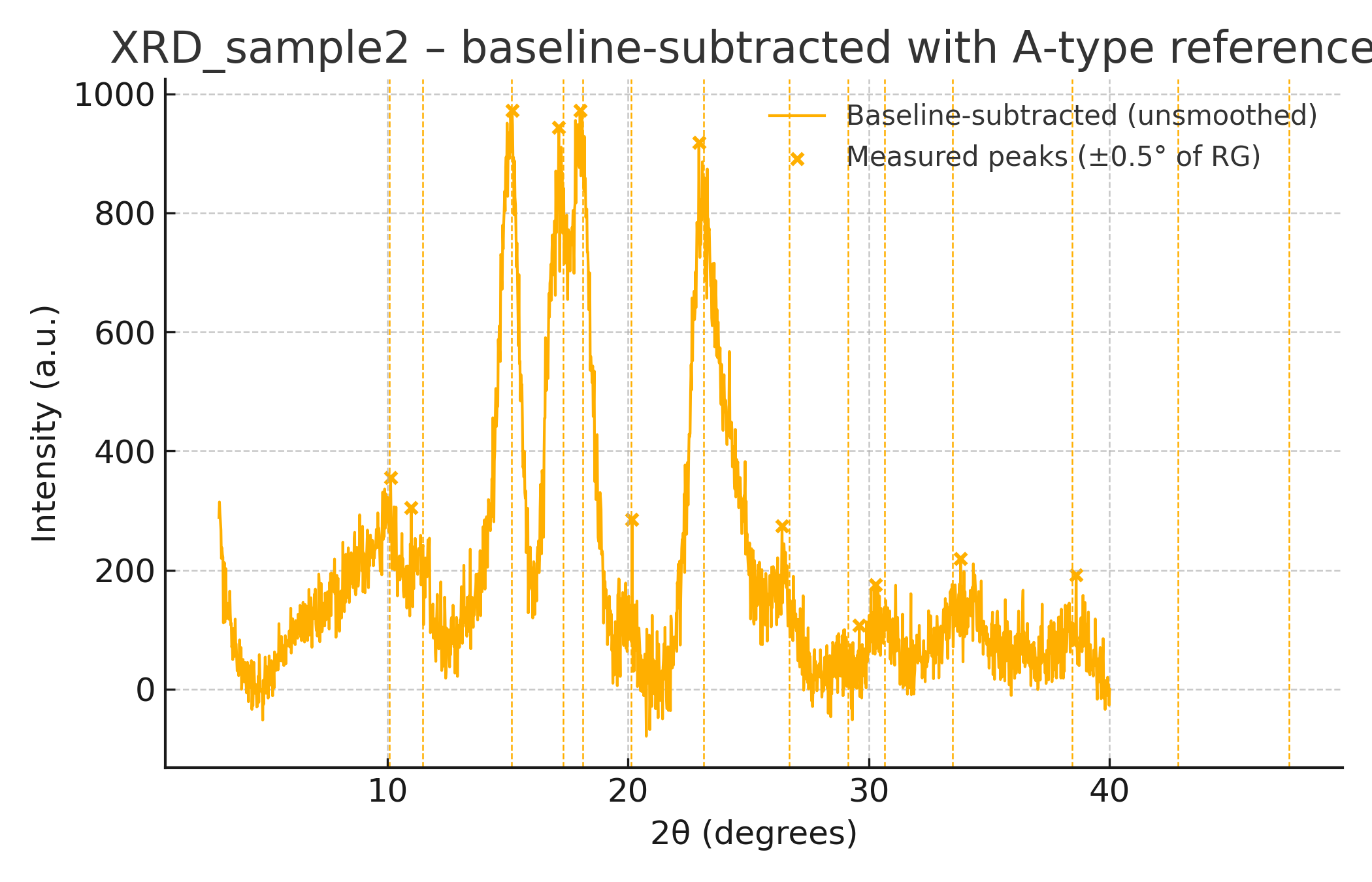

Supplement: Supplementary file 1 [file foods-14-04130-s001.zip › Supplementary data (XRD Analysed data)/Supplementary data (XRD Analysed data)/ZN19/XRD_sample2_corrected_peaks.png]

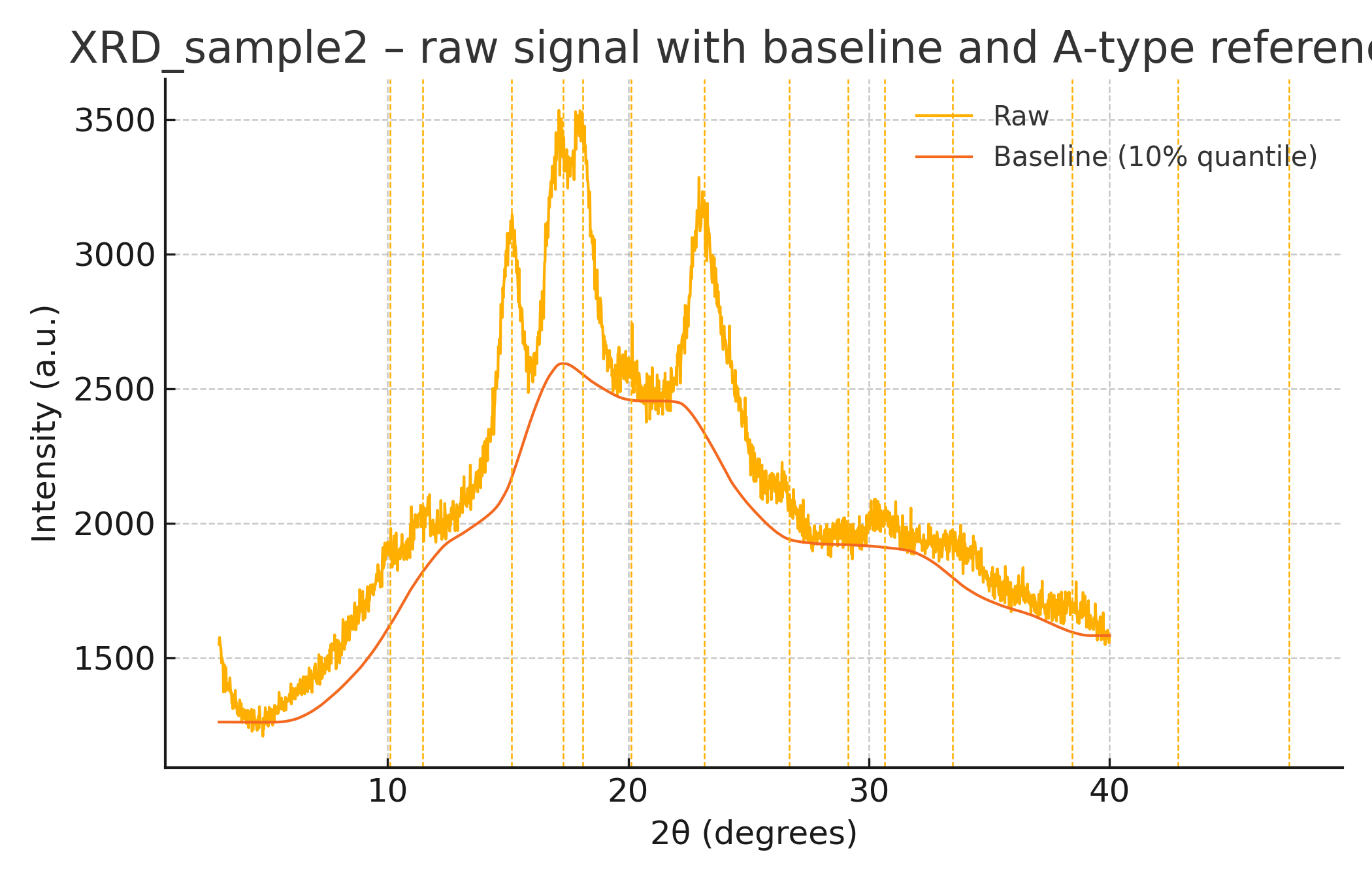

Supplement: Supplementary file 1 [file foods-14-04130-s001.zip › Supplementary data (XRD Analysed data)/Supplementary data (XRD Analysed data)/ZN19/XRD_sample2_raw_baseline.png]

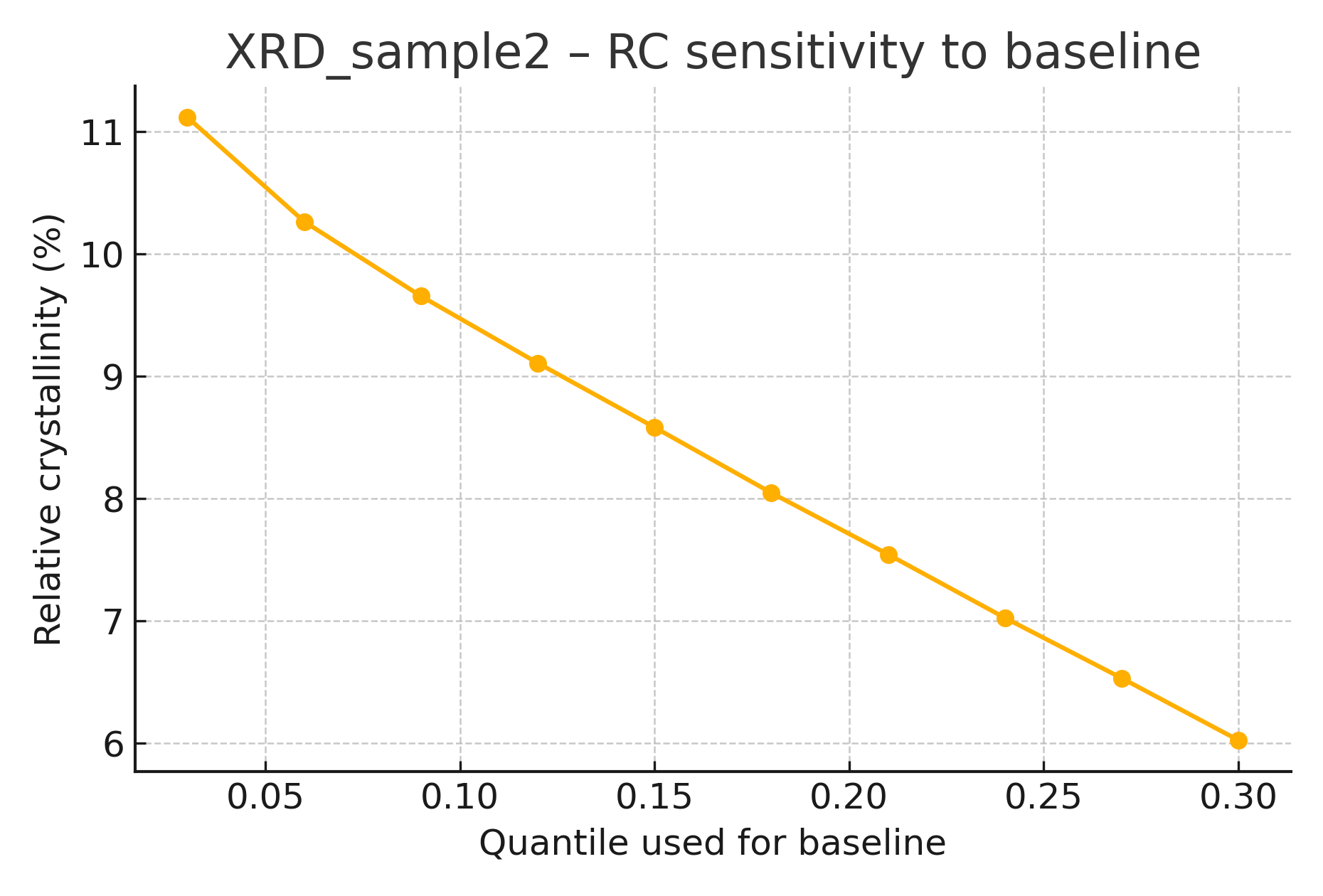

Supplement: Supplementary file 1 [file foods-14-04130-s001.zip › Supplementary data (XRD Analysed data)/Supplementary data (XRD Analysed data)/ZN19/XRD_sample2_RC_sensitivity.png]

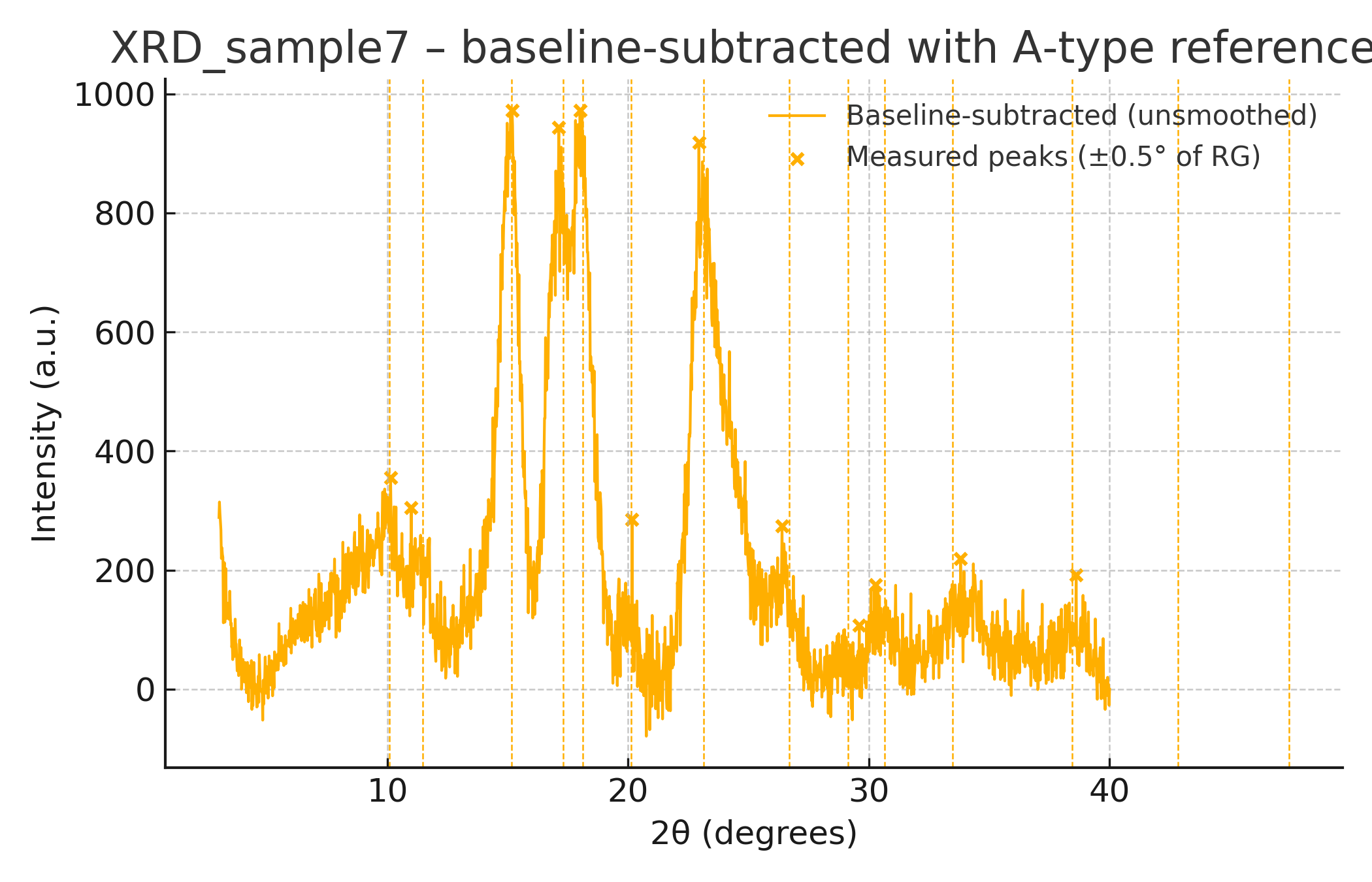

Supplement: Supplementary file 1 [file foods-14-04130-s001.zip › Supplementary data (XRD Analysed data)/Supplementary data (XRD Analysed data)/ZN65/XRD_sample7_corrected_peaks.png]

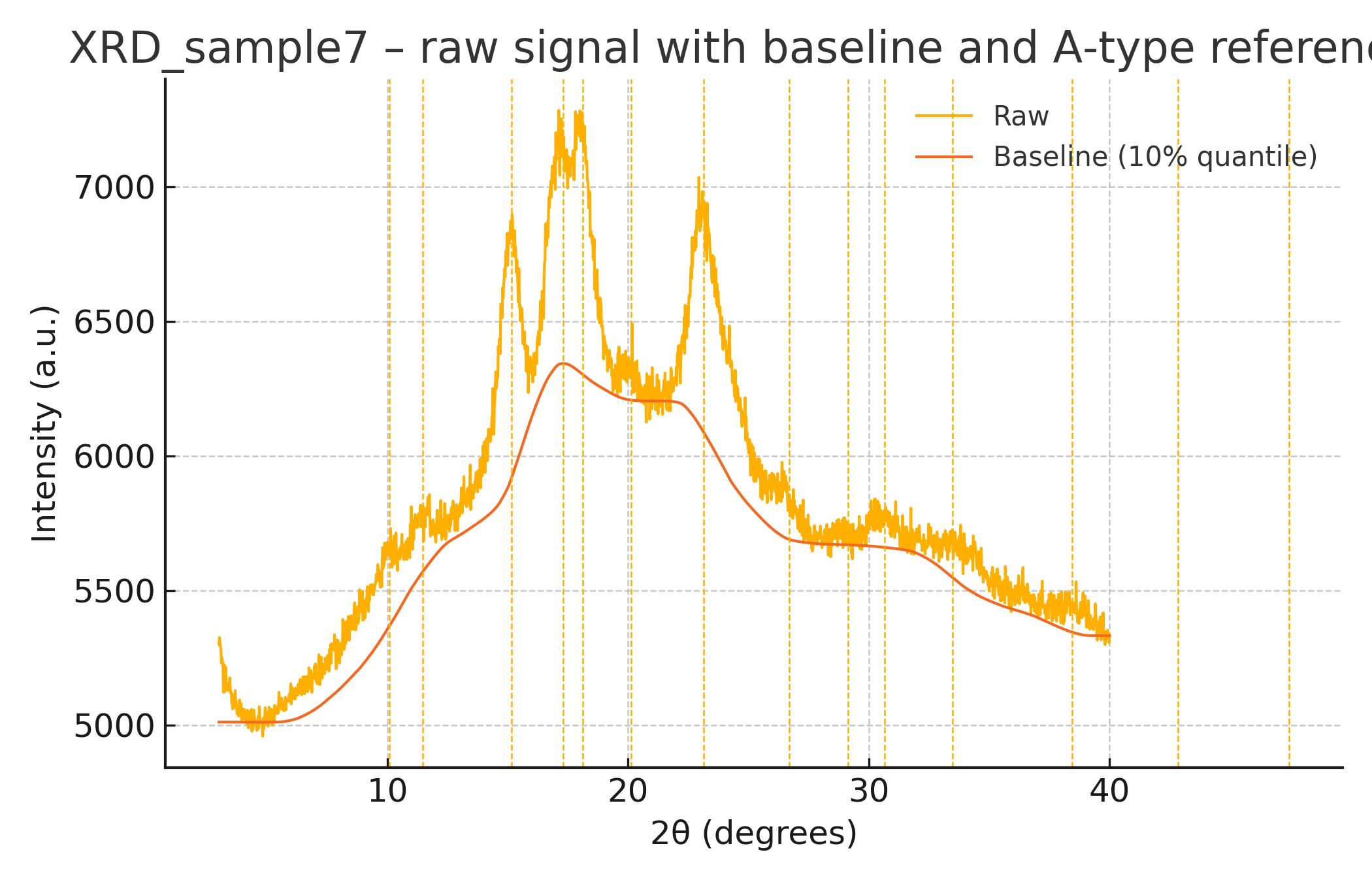

Supplement: Supplementary file 1 [file foods-14-04130-s001.zip › Supplementary data (XRD Analysed data)/Supplementary data (XRD Analysed data)/ZN65/XRD_sample7_raw_baseline.png]

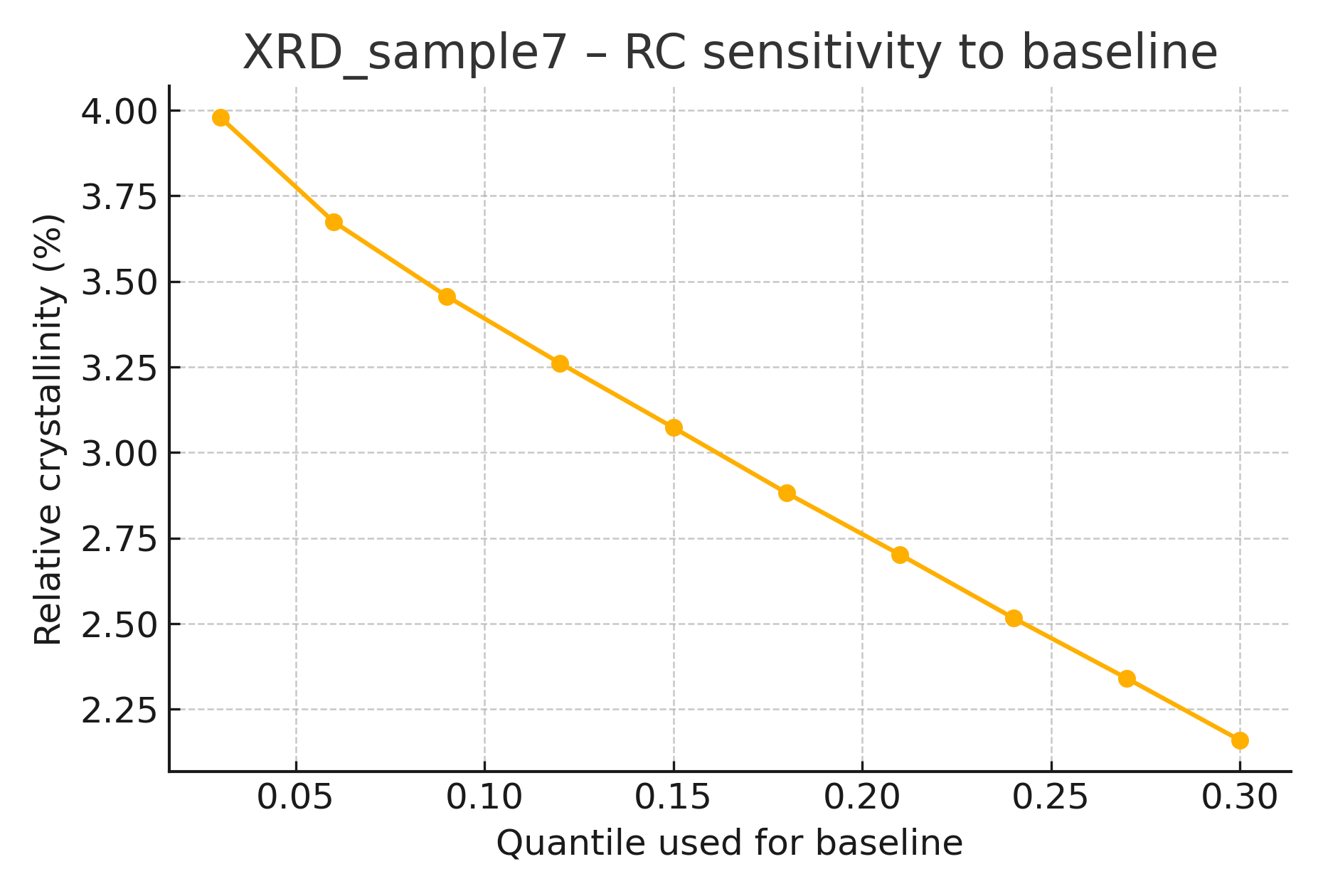

Supplement: Supplementary file 1 [file foods-14-04130-s001.zip › Supplementary data (XRD Analysed data)/Supplementary data (XRD Analysed data)/ZN65/XRD_sample7_RC_sensitivity.png]
